# Bacterial genome engineering using CRISPR-associated transposases

**DOI:** 10.1038/s41596-023-00927-3

**Published:** 2024-01-12

**Authors:** Diego Rivera Gelsinger, Phuc Leo H. Vo, Sanne E. Klompe, Carlotta Ronda, Harris H. Wang, Samuel H. Sternberg

**Affiliations:** 1 Department of Biochemistry and Molecular Biophysics, Columbia University, New York, NY, USA.; 2 Department of Systems Biology, Columbia University, New York, NY, USA.; 3 Department of Molecular Pharmacology and Therapeutics, Columbia University, New York, NY, USA.; 4 Department of Pathology and Cell Biology, Columbia University, New York, NY, USA.; 5 Present address: Vertex Pharmaceuticals, Inc, Boston, MA, USA.; 6 Present address: Department of Genomes and Genetics, Institut Pasteur, Paris, France.; 7 Present address: Innovative Genomics Institute, University of California Berkeley, Berkeley, CA, USA.

## Abstract

Clustered regularly interspaced short palindromic repeats (CRISPR)-associated transposases have the potential to transform the technology landscape for kilobase-scale genome engineering, by virtue of their ability to integrate large genetic payloads with high accuracy, easy programmability and no requirement for homologous recombination machinery. These transposons encode efficient, CRISPR RNA-guided transposases that execute genomic insertions in *Escherichia coli* at efficiencies approaching ~100%. Moreover, they generate multiplexed edits when programmed with multiple guides, and function robustly in diverse Gram-negative bacterial species. Here we present a detailed protocol for engineering bacterial genomes using CRISPR-associated transposase (CAST) systems, including guidelines on the available vectors, customization of guide RNAs and DNA payloads, selection of common delivery methods, and genotypic analysis of integration events. We further describe a computational CRISPR RNA design algorithm to avoid potential off-targets, and a CRISPR array cloning pipeline for performing multiplexed DNA insertions. The method presented here allows the isolation of clonal strains containing a novel genomic integration event of interest within 1–2 weeks using available plasmid constructs and standard molecular biology techniques.

## Introduction

The field of biology has been revolutionized by the discovery of adaptive immune systems encoded by clustered regularly interspaced short palindromic repeats (CRISPR) and CRISPR-associated (Cas) genes, and the subsequent harnessing of CRISPR–Cas systems for genome engineering. In particular, CRISPR–Cas-based genetic manipulations have been very efficiently applied to several model (i.e., *Escherichia coli*) and nonmodel organisms across all domains of life^1–4^, deepening our understanding of the biology underlying these organisms. CRISPR–Cas systems encode a diverse repertoire of RNA-guided CRISPR-associated effector nucleases that perform interference on invading mobile genetic elements. These programmable endonucleases can be directed to target nearly any DNA or RNA sequences for interference by complexing with a noncoding CRISPR RNA (crRNA, also referred to as a guide RNA), and have been efficiently repurposed as powerful genome editing tools^5–9^. The most established of these biotechnologies, CRISPR–Cas9, allows targeted cutting of double-stranded DNA and has vastly expanded the eukaryotic genome engineering toolkit^10,11^. Despite their bacterial origins, though, conventional CRISPR-based approaches have not drastically changed the landscape of bacterial genome engineering due to various limitations, including cytotoxicity^12^.

Many bacterial engineering applications have instead utilized recombineering, a common method based on homologous recombination (HR) between a genomic DNA (gDNA) sequence and a synthetic, user-provided donor molecule containing the desired DNA insert flanked by homology arms^13,14^. In recent years, CRISPR–Cas9 has been combined with recombineering to provide counterselection against unedited cells via targeted cleavage of the wild-type allele, thereby enabling programmable scarless editing without the need for drug marker selection^15–19^. Recombineering strategies, while often effective, typically require the introduction of exogenous recombination machinery (e.g., Lambda red system), and can yield low efficiency, particularly for the insertion of multi-kilobase DNA payloads^20^. Additionally, recombineering often translates poorly between diverse target species due to host specificity of exogenous recombination proteins^21^.

Recent advances in other areas of synthetic biology and genome engineering have provided novel and exciting avenues to further our understanding of bacterial cellular biology, pathogenicity and functional genomics^22^. These advances have led to the use of bacteria as diagnostic and therapeutic agents targeting a variety of diseases^23^, as well as their use as ‘biofactories’ for industrial production of biofuels and beneficial small molecules^24^. Many of these applications require genomic insertion of customized DNA payloads (i.e., ‘knock-ins’), which allow for stable maintenance of desired expression cassettes at predictable copy numbers and reduced metabolic burden, without the need for drug marker selection and overcoming the population heterogeneity typically associated with plasmid-based expression^25^. However, existing transposase and recombinase platforms commonly applied for DNA insertion, such as Cre recombinase or Tn*7* transposase, recognize fixed target sequences and are thus not readily programmable^26–29^. Integration-based systems, such as Tn*5* or Mariner transposase systems, can be used as an alternative; however, they exhibit little to no target specificity and catalyze insertion into random genomic sites^30,31^, which is undesirable for systematic and controllable strain engineering programs.

**Fig. 1 |:**
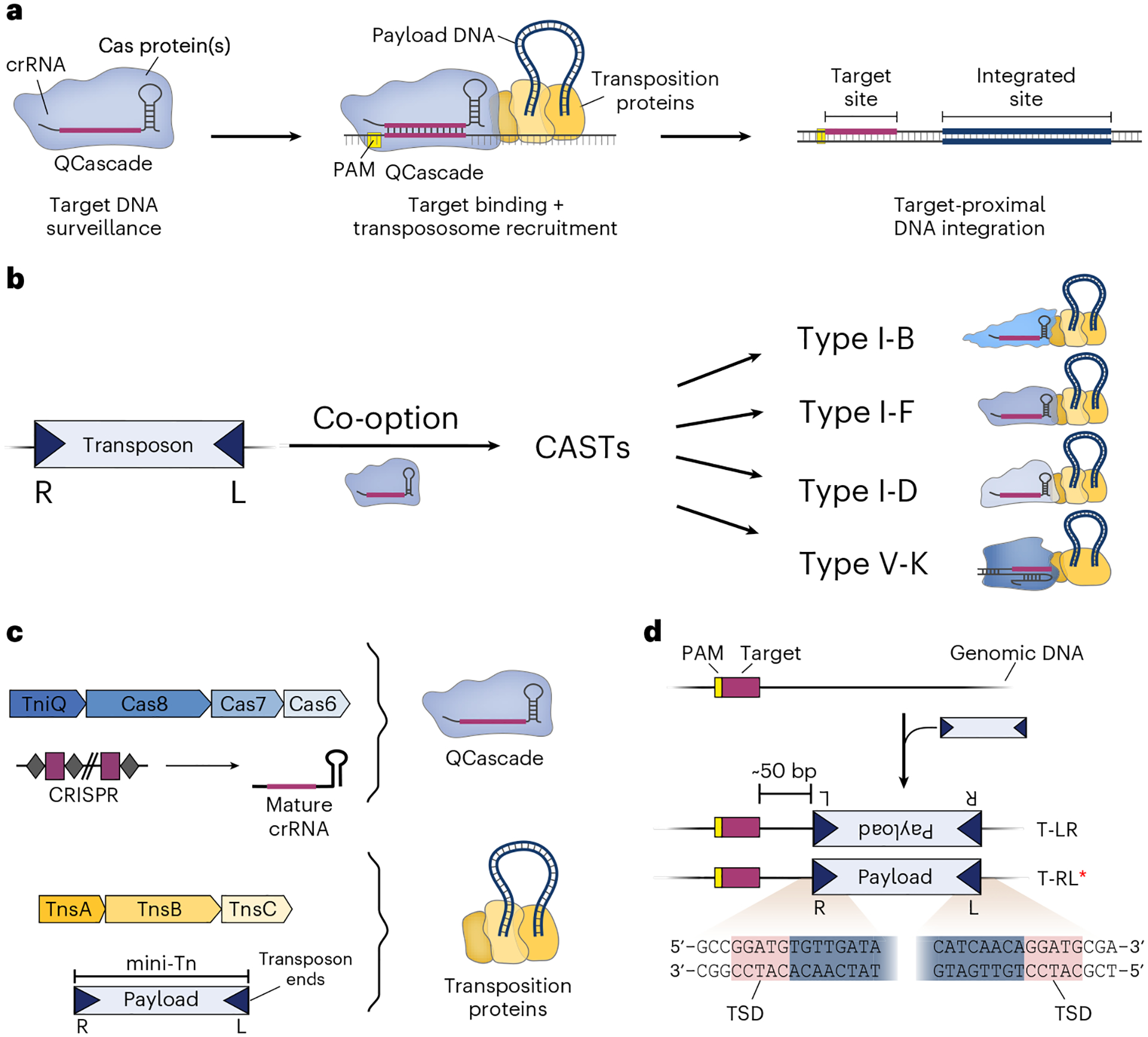
Overview of CASTs. **a**, A simplified schematic of the general mechanism of RNA-guided DNA transposition. CRISPR–Cas effector complexes, consisting of a mature crRNA and one or more Cas proteins, recognize and bind genomic target sites using RNA–DNA complementarity. Subsequent recruitment of transposase proteins in complex with the donor DNA (mini-Tn, in blue) leads to integration of the mini-Tn at a fixed distance downstream of the target site. The mini-Tn can be customized with user-defined payloads. **b**, Tn*7*-like transposons have co-opted at least four different families of nuclease-deficient CRISPR–Cas systems during CAST evolution: Type I-B, I-D, I-F and V-K. **c**, Main components of QCascade and transposition protein complexes. Top: required components for RNA-guided DNA integration using Type I-F CASTs. The DNA binding complex, QCascade, consists of TniQ, multiple Cas proteins, and a mature crRNA that is processed by Cas6. Bottom: TnsA and TnsB catalyze DNA excision and integration chemistry, aided by the mediator ATPase, TnsC. Mini-Tn substrates must be flanked by transposon right (R) and left (L) ends. **d**, DNA insertions occur ~50 bp downstream of the target site in one of two possible orientations, defined by which transposon end (T-LR and T-RL) is closest to the target site. T-RL products (*) are preferentially generated by Type I-F CASTs, and products exhibit hallmark 5-bp TSDs.

To overcome these limitations, we recently described a powerful new addition to the genome engineering toolbox, which exploits CRISPR-associated transposases (CASTs) to achieve highly efficient and targeted DNA integration of large kilobase-scale payloads^32,33^ (Fig. 1a). Since their initial bioinformatic discovery^34^, we and others have harnessed CAST systems for a range of engineering applications in diverse microbial species^32,35–38^ Our approach combines the ease of programmability of CRISPR–Cas systems with the efficient chemistry afforded by transposase enzymes, and allows targeted DNA insertions without requiring DNA double-strand breaks or recombination machinery. This protocol describes the CAST-mediated genetic editing of bacteria that exploits the ability of CAST systems to integrate payloads with high efficiency, perform multiplexed insertions with multiple guide RNAs, and mobilize payloads ranging from less than 1 kb to more than 10 kb in size.

### Mechanism and development of Type I-F CASTs

CRISPR-associated transposons are evolutionarily diverse, since they arose from at least four independent exaptation events in which Tn*7*-like transposons repurposed nuclease-deficient CRISPR–Cas systems from Type I-B, Type I-D, Type I-F, or Type V-K^33,39–44^ (Fig. 1b). All CAST systems adopt the conserved DDE family TnsB transposase, which performs the concerted strand transfer reactions during transposition, alongside common accessory factors, including TnsC and TniQ. However, the molecular basis of DNA targeting differs: Type I CAST systems employ a multisubunit RNA-guided DNA binding complex, called Cascade, for target selection, whereas Type V-K CAST systems employ the single-effector protein Cas12k (refs. 33,39,40,42). Importantly, other mechanistic parameters strongly differ between CAST systems, including the number of molecular components, the purity of insertion products, the genome-wide fidelity, and on-target efficiency^33,37,39,42,45–47^. Due to key advantages reported for Type I-F CAST systems^32^, such as high specificity and purity of integration products, we further improved the technology behind these systems and provide in this protocol technical details and guidelines for their use in bacteria. However, numerous recent studies describe both mechanistic and technological advances of Type V-K CAST systems^45,47–49^.

Natural CRISPR-associated transposons are bounded by conserved transposon left (L)- and right (R)-end sequences. Therefore, genetic payloads for site-specific genomic insertion end by engineered CAST systems must also be encoded within a mini-transposon (mini-Tn) context bounded by the same sequence features. Type I-F CAST systems encode two molecular machineries for directing and catalyzing RNA-guided DNA transposition: an RNA-guided DNA targeting complex known as TniQ-Cascade (hereafter QCascade), which comprises a crRNA guide and protein components TniQ, Cas8, Cas7 and Cas6 (refs. 33,40), and the heteromeric transposase complex TnsABC, which consists of the TnsA endonuclease, the TnsB transposase, and the TnsC ATPase^33,50^ (Fig. 1c). QCascade uses a 32-nt guide sequence to bind 32-bp DNA target sites flanked by a 5′-CN-3′ protospacer adjacent motif (PAM) sequence^33,40,41^, leading to the integration of the mini-Tn at a fixed distance of ~50 bp downstream of the target site (integration site) defined by the molecular footprint of associated transposition proteins^33,46,51^ (Fig. 1d). Importantly, the DNA integration event does not disrupt the target site itself, leaving open the possibility that constitutive expression of CAST machinery could lead to iterative rounds of targeting and DNA insertion. However, these tandem insertions are rarely generated because of a feature intrinsic to Tn*7*-like transposons known as target immunity (see below)^33,42,50,52–55^.

Type I-F CASTs generate simple insertion products through a non-replicative cut-and-paste reaction, whereas Type V-K CASTs, which lack the TnsA endonuclease, generate cointegrate products through a replicative mechanism^46,56,57^. Transposition products feature hallmark target-site duplications (TSDs) flanking the inserted payload, in which 5 bp of genomic sequence is precisely duplicated (Fig. 1d). Orientation control is another key feature of RNA-guided transposition. Although the left and right ends feature repetitive TnsB binding sites and are reminiscent of the terminal inverted repeats characteristic of other transposon families, the positioning of these binding sites is distinct on both ends, leading to a striking asymmetry that favors polarized insertions. Type I-F CASTs generate integration products in both of two possible orientations, referred to as T-LR and T-RL (Fig. 1d), but the T-RL products are highly preferred at ratios typically over 90%, depending on the crRNA and CAST system^32,33,41^. Thus, this bias should be taken into account when utilizing payloads where expression is orientation dependent (i.e., promoter capture).

In our early work, we discovered and characterized RNA-guided DNA integration using a representative Type I-F CRISPR-associated transposon from *Vibrio cholerae*, which was assigned the transposon identifier Tn6677 (refs. 33,58), previously referred to as VchINTEGRATE (VchINT), and is hereafter referred to as VchCAST to reconcile prior nomenclature choices in the literature. Genomic insertions were performed in *E. coli* by cotransforming cells with three separate vectors, a pDonor encoding the mini-Tn, a pQCascade encoding the TniQ-Cascade complex, and a pTnsABC encoding the heteromeric TnsABC transposase. Using this system, we reported efficiencies of 40–60% when using genetic payloads of 980 bp^33^. We also readily achieved integration of DNA payloads up to 10 kb in size and detected integration at 24 genomic target sites tiled across the *E. coli* genome, highlighting the robust programmability of the system. We adopted a high-throughput sequencing approach, termed transposon-insertion sequencing (Tn-seq)^31,33^, to unbiasedly query the genome-wide specificity of integration products, and found that VchCAST exhibited remarkable fidelity, with the majority of crRNAs displaying >95% on-target accuracy and many exceeding 99%. This represents a major advance given that much lower fidelity and Cas12k-independent integration activity has been reported for Type V-K CAST systems, including ShCAST and ShoCAST (formerly ShoINT)^32,42,47,49^, indicating that some CAST systems retain the ability to undergo random, untargeted transposition. Importantly, recent work from Kleinstiver and colleagues has reported some level of improvement in the specificity of Type V-K CAST systems, as well as engineered systems known as HELIX, which reduce the frequency of cointegrate product formation^45^.

**Fig. 2 |:**
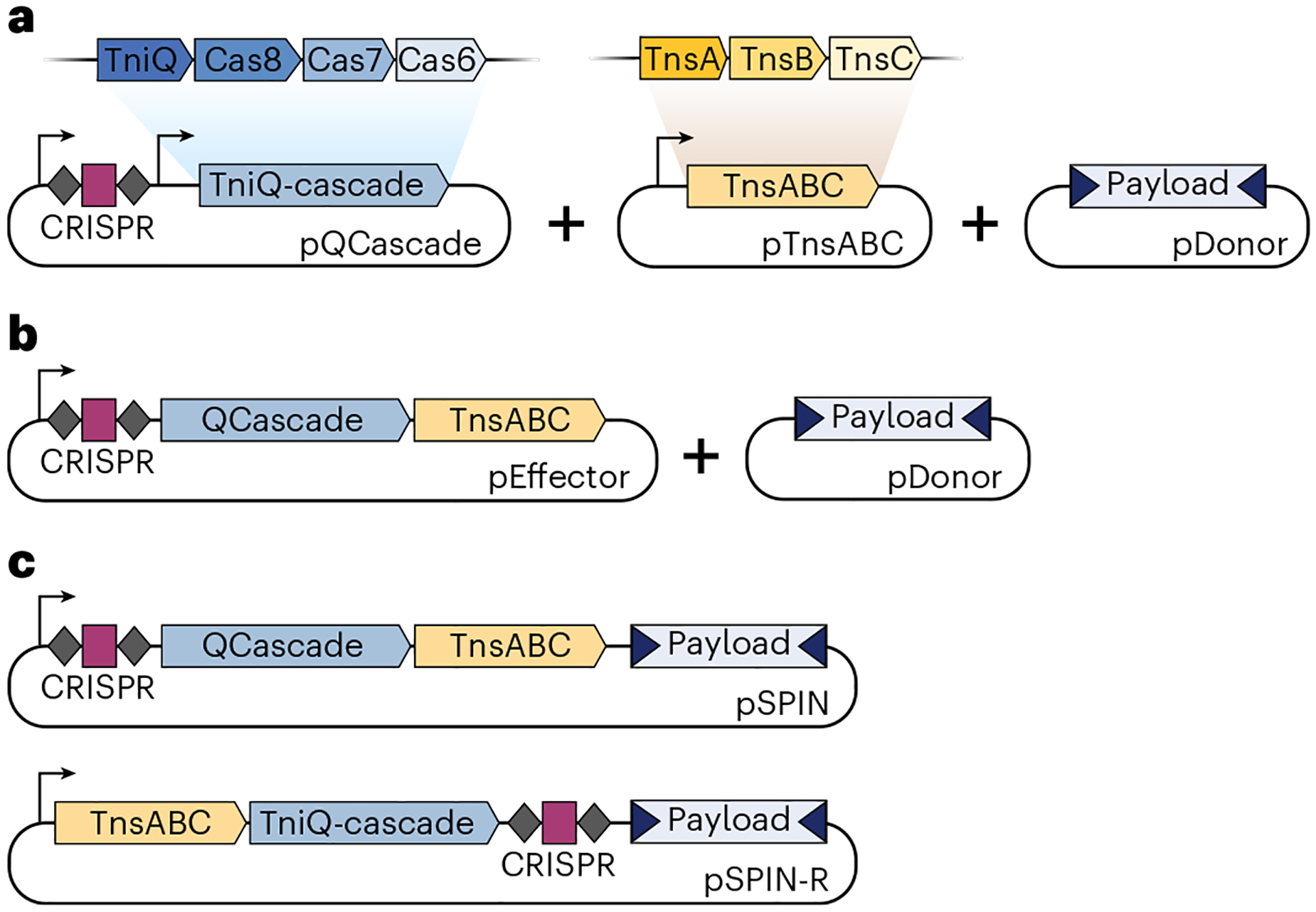
Architecture of VchCAST vector constructs. **a**, A three-plasmid system for CAST expression includes a vector that encodes the crRNA and QCascade components (pQCascade), a vector that encodes TnsABC (pTnsABC) and a final vector that harbors the payload flanked by transposon ends (pDonor). **b**, A two-plasmid system consists of an expression vector vector with single promoter driving expression of crRNA, QCascade and TnsABC (pEffector), and a second vector harboring the mini-Tn (pDonor). **c**, Two different variants of highly efficient single-plasmid integration vectors (pSPIN) are available. Top: pSPIN with a single promoter driving the expression of the crRNA, QCascade, and TnsABC followed by the mini-Tn. Bottom: pSPIN-R that reduces self-targeting insertions, in which the CRISPR array is relocated just upstream of the mini-Tn.

While our initial study introduced VchCAST as a promising genome engineering platform, the requirement for three separate plasmids encoding multiple expression cassettes limits construct customization and delivery, preventing broader application of the system. To address this, we designed a streamlined expression cassette termed pEffector, which expresses all necessary protein and RNA components from a single polycistronic promoter, and then combined this with the mini-Tn genetic payload to generate single-plasmid integration vectors, termed pSPIN^32^ (Fig. 2). These vectors considerably simplified CAST delivery to diverse bacteria, led to integration efficiencies approaching 100% after promoter and backbone optimization, and enabled straightforward plasmid curing to remove the CAST system from cells after the desired genomic insertion was introduced^32^. Parallel efforts from Doudna and colleagues similarly highlighted the versatility, efficiency, and specificity of single-plasmid Type I-F CAST systems for bacterial genome engineering, termed VcDART^37^. Interestingly, for large genetic payloads (~10 kb) we found that VchCAST exhibited higher integration efficiencies when cells were incubated at temperatures below 37 °C, without a detectable change in genome-wide specificity^32^. Although the molecular basis for this temperature effect is not yet fully understood, it provided an accessible strategy to increase the yield of edited cells with large genetic payloads and may be worth exploring further for certain downstream applications.

### Multiplexed insertions

**Fig. 3 |:**
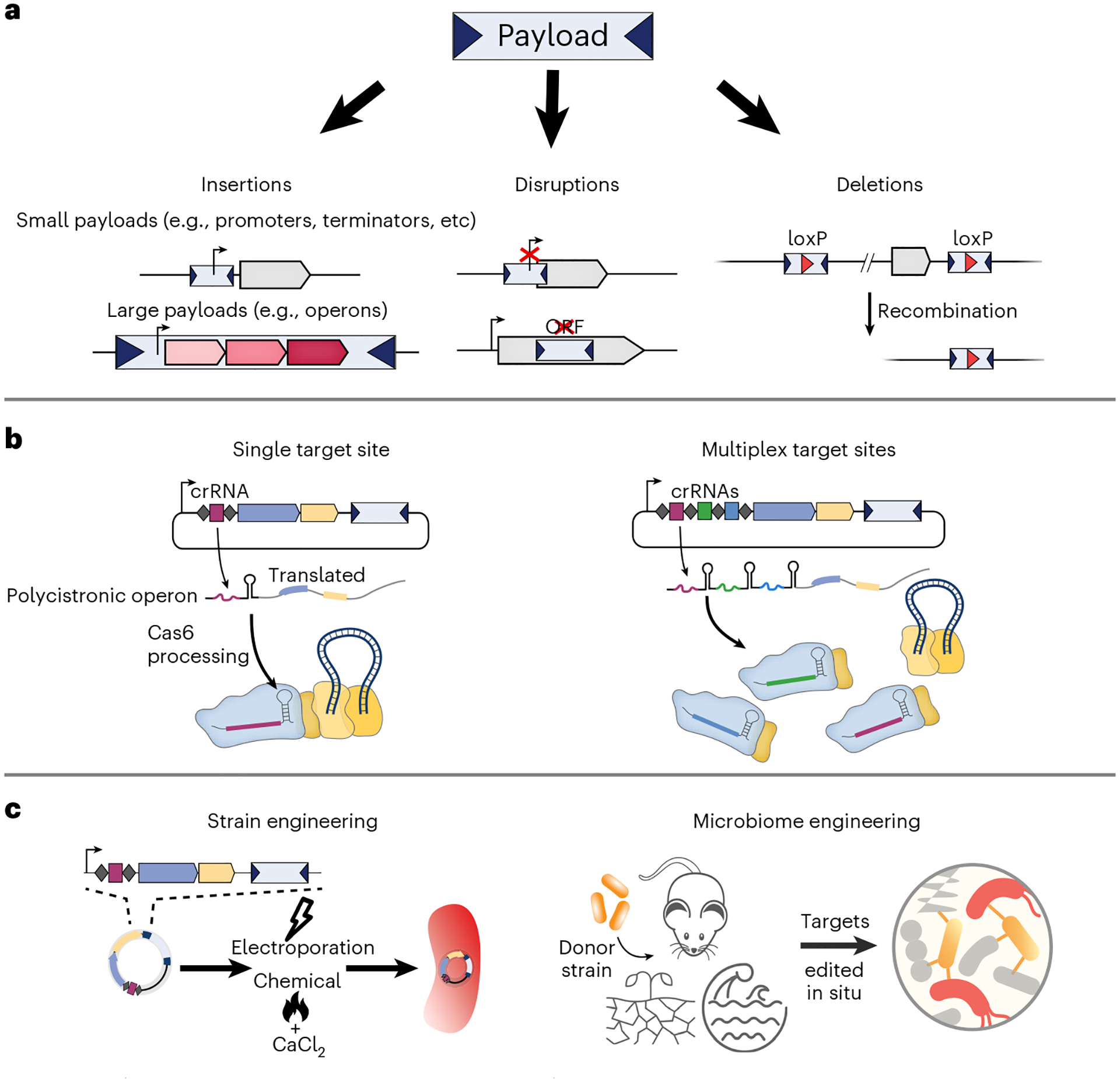
Possible applications of CAST systems for microbial engineering. **a**, A schematic depicting the range of genetic modifications that can be generated with CASTs, which include insertions of either small payloads (e.g., promoters, terminators, repressors, etc.) or large payloads (e.g., genes or metabolic operons), disruption of the ORFs via insertion into promoters or within a gene, or programmed deletions via Cre-based recombination of inserted loxP sites. Functional insertions can also be generated that simultaneously disrupt endogenous genes. **b**, The modifications in **a** can be made at single target sites (left) or multiple target sites using multispacer CRISPR arrays (right). crRNAs are first transcribed as a precursor transcript that is processed by Cas6 to form mature crRNAs. **c**, RNA-guided insertions can be used to engineer single-strain isolates using typical transformation methods (left) or to precisely modify complex microbial communities in diverse environments (e.g., gut, soil and/or aquatic microbiomes) via conjugative delivery (right).

Site-specific transposases and integrases such as Tn*7* and Bxb1, which perform efficient genomic integration, cannot be easily reprogrammed. Unlike these enzymes, CAST systems can be easily programmed to direct the insertion event at a single or multiple new user-defined target sites, when using multiple guide RNAs^32,35^. For example, we have shown that multispacer CRISPR arrays are efficiently processed into multiple crRNAs in bacteria (Fig. 3a,b), leading to multiplexed and simultaneous insertion of the same DNA payload at up to three target sites, enabling rapid generation of insertional knockouts^32,35^. Additionally, by encoding a loxP sequence within the mini-Tn payload, we combined VchCAST with Cre recombinase to mediate seamless, programmed deletions of large genomic sequences^32^ (Fig. 3a). Yang and colleagues successfully employed multispacer CRISPR arrays alongside single-spacer arrays targeting multicopy genomic loci, in a strategy that produced *E. coli* strains containing up to ten genomic insertions of a glucose dehydrogenase expression cassette^59^. Finally, the use of pooled guide RNA libraries with both Type I-F and Type V-K systems across a population of cells enables efficient disruption of a subset of genes of interest^36,51^, with analogous results to the use of Cas9 and guide RNA libraries for genetic screening experiments in eukaryotic cells.

### Homologous and orthogonal Type I-F CAST systems

CRISPR–Cas systems are highly diverse, and bioinformatic and experimental mining efforts over the years have repeatedly uncovered new variants that offer advantages for technology development. Thus, we and others have been similarly motivated to explore and develop additional CAST elements for programmable RNA-guided DNA integration^41,43,60^. We recently employed a bioinformatics pipeline to identify hundreds of Type I-F CAST systems in sequenced bacterial genomes, and then experimentally characterized 18 new systems that exhibited integration activity in *E. coli*^41^. These homologs display interesting behaviors when compared with VchCAST, including intriguing modularity between the CRISPR and transposase components, the presence of additional targeting proteins or protein–protein fusions and unique recognition of transposon end sequences. We identified four additional CAST systems that were capable of highly efficient and accurate DNA insertions, forming a suite of high-value CAST systems for bacterial genome engineering alongside VchCAST. Importantly, these five systems are completely orthogonal: the transposon end sequences from each system are effectively invisible to the transposase machineries from the others, such that genetic payloads encoded within the mini-Tn can be selectively acted upon by only the cognate CAST machinery. This orthogonality could allow consecutive insertions to be generated within a focused genomic region of interest, without the inhibitory consequences of the target immunity pathway that normally precludes tandem insertions at the same site^55^. Collectively, these diverse CAST systems open up the possibility of rapid and iterative engineering of target strains, as well as enabling the selection of a CAST system of choice for applications within new bacterial species of interest.

### Applications of CAST systems

CAST systems provide a simple yet powerful platform for inserting DNA payloads ranging from hundreds to thousands of base pairs in size (Fig. 3a). Smaller payloads generally include regulatory elements, such as promoters, to drive inactive genes, and terminators, to decouple transcription and translation, as well as other types of regulatory elements (Fig. 3a). On the contrary, larger payloads can harbor entire operons that enable metabolic rewiring within a target bacterial strain (Fig. 3a). As transposons are ubiquitous selfish genetic elements that often mobilize across various hosts, their applications across diverse bacterial species and strains that have never been genetically modified before is particularly appealing (Fig. 3c). For example, VchCAST, which was initially characterized in standard *E. coli* strains, has been successfully used by us and others to mediate robust genomic integration in multiple strains, including recombination-deficient *E. coli* strains^32,33,35,42^, multiple species of *Klebsiella* and *Pseudomonas*^32,37^, and in *Tatumella citrea*^59^ (Fig. 3c). In addition, the design of pSPIN vectors, which encode all necessary molecular components, has opened the door to seamless delivery of CAST systems between bacteria via conjugation (i.e., the exchange of genetic material through direct cell contact). An exciting application of CASTs in various nonmodel bacteria is the multiplexed targeted gene knock outs, all at one time, using a multispacer CRISPR array (Fig. 3a,b). This approach can uncover the functions of unknown genes essential to environmental conditions and perturbations, akin to genome-wide transposon mutagenesis libraries but with a more targeted resolution^31^. CASTs thus hold the promise to uncover the dearth of known biological functions that exist in the genomes of bacteria.

In addition to rapidly engineering bacterial species through loss-of-function insertional mutagenesis, the integrative capacity of CAST systems can be further exploited for the stable genomic integration of single transgenes and potentially even entire operonic pathways, with high efficiency and specificity^32^ (Fig. 3a). In this regard, Yang and colleagues utilized the VchCAST system to engineer and rapidly optimize strains for the biosynthesis of key industrial compounds by performing multiplexed targeting. In particular, they achieved multicopy genomic integration of synthesis pathways while also disrupting undesired host degradation pathways^35^ (Fig. 3b). This strategy produced modified strains displaying more robust and stable generation of compounds of interest compared to plasmid-based expression.

While engineering individual strains is often desirable (Fig. 3c), microorganisms naturally exist within consortia in ecosystems, as members of complex communities with other bacteria, archaea and eukaryotes. Thus, studying bacteria in isolation limits our understanding of their natural physiology. We and others have developed a foundation to use CAST systems for in situ engineering of target species within complex bacterial communities^32,37^, including species that have so far been challenging to edit using existing technologies (Fig. 3c). If applied to microbial communities, the previously described CAST-based genetic engineering approaches can potentially bridge the gap between our functional understanding of a few cultivable microbes and the overwhelming diversity of uncultivated microbes found in all ecosystems. In addition, integrating desired genetic payloads into microorganisms will increase the range of possibilities for probiotic-based therapies. Future applications focusing on broad-host-range CAST expression vectors and transient delivery, will vastly expand the toolkit for microbial engineering.

### Comparisons with existing methods

Transposases and integrases are versatile and pervasive genes across all domains of life^61^ and form the foundation for many existing technologies that mediate large DNA insertions into bacterial genomes. For example, systems such as the ICEBs1 integrative element, Cre–loxP recombinase system or the Tn*7* transposon, have been used for chromosomal integration of exogenous genes and pathways in diverse bacteria^28,62,63^. However, these systems are only able to recognize fixed, system-specific target sequences that must already be present in the target strain before editing or be installed separately using an orthogonal method^28,62,63^. Other transposon systems, such as Mariner or Tn*5*, facilitate genomic insertions with minimal sequence specificity^64–67^, and therefore enable genome-wide transposon mutagenesis or high-throughput integration screens^31,66,68–70^. However, applications requiring targeted insertions at desired loci cannot effectively make use of such nonspecific integrative systems, and clones containing inserts at desired locations must first be identified through additional steps, such as whole-genome sequencing. In this case, platforms with native target programmability, such as recombination-based strategies and CAST systems, are preferable to simplify and accelerate engineering workflows.

Compared with recombineering-based technologies, CAST systems offer several key advantages. Recombineering efficiency is generally low (less than 1 in 10^3^–10^4^) (ref. 71) and typically requires the selection of a co-integrating selectable marker^72^ or CRISPR–Cas-mediated counter selection of unedited alleles^17^, making it inadequate for multiplexing. In contrast, CAST systems mediate insertions of large, multi-kilobase DNA payloads with nearly 100% efficiency without requiring any selection for integration events^32,37^, which can be advantageous when the use of drug markers is undesired. While the requirement for fixed transposon end sequences flanking the insert of interest does preclude scarless insertions, the absence of a recombination-based process for CAST insertions overcome the need for homology arms. In contrast, recombineering-based methodologies rely on donor DNA molecules harboring arms homologous to the sequences directly flanking the targeted insertion site, as well as unique selection marker cassettes, though notable advancements have facilitated marker removal^73,74^, Therefore, cloning of such donor molecules quickly becomes time and labor intensive to generate, especially for multiplexed editing experiments^75^. Thus, for applications such as those involving insertional mutagenesis or intergenic integration of multi-kilobase payloads, the use of CAST systems is recommended.

### Limitations of CAST systems

Mobilization of the mini-Tn by CAST systems requires recognition of and binding to conserved transposon end sequences, classically referred to as the transposon left and right ends, by the transposase machinery. Thus, any DNA payload of interest must be bounded by these end sequences as part of the entire functional mini-Tn (Fig. 1). The transposon ends contain asymmetrical transposase binding sites which play a role in the orientation of integration (T-RL versus T-LR). The transposon ends do not impede transcription, and they can also be engineered to encode functional protein linker sequences for precise in-frame knock-in applications^76^. Transposase-mediated insertions predominantly occur 49 bp downstream of the 3′-end of the 32-bp target site. However, the exact distribution of distances sampled across a population of cells depends on local sequence features, meaning that a certain degree of variability in the selection of the precise integration site has been reported^33,41^. This lack of single-nucleotide specificity may reduce effectiveness of certain applications, such as promoter capture or in-frame insertions. We recently employed library-based experiments to further investigate VchCAST target-site specificity and uncovered novel TnsB transposase sequence preference, which will be instrumental in enabling nucleotide-level control over integration products in future applications^76^. Additionally, Type I-F CAST systems generate insertions in two possible orientations (Fig. 1), although T-RL products are preferred by ratios typically exceeding 100:1, especially for recently reported homologous CAST systems^38,41^. Thus, engineering applications that require scarless insertions should preferentially utilize HR-based methods, which exhibit greater payload constraints and suffer from low efficiency, while enabling editing without transposon end requirements.

CAST systems generate insertions at a fixed distance downstream of the genomic site targeted by a guide RNA. Therefore, the target sequence is not disrupted upon integration, such that persistent expression of the enzymatic machinery could potentially lead to repeated rounds of insertion, resulting in multiple transposon copies in tandem. However, a target immunity pathway that depends on molecular interactions between TnsB and TnsC^46,77^ inhibits multiple transposon insertions at the same genomic site. Target immunity is most effective for insertions directly adjacent to an existing transposon insertion and gradually decays at further distances, with ~20% of the expected activity being restored at a 5-kb distance^32,46^. While target immunity strongly inhibits tandem transposon insertions, it has been observed that these undesired products are nevertheless generated at low levels, particularly in scenarios involving strong constitutive expression of the system over extended periods of time^55^. Moreover, self-targeting integration events have also been observed (Box 1). In this case, QCascade targets the spacer sequence within the CRISPR array itself during RNA-guided transposition, resulting in insertion events within the expression cassette that can inactivate the CAST system^32^. Another alternative integration product that CAST systems generate are cointegrates, which consist of duplicated transposon copies and genomic insertion of the vector backbone (Box 1). While rare in Type I-F CASTs, cointegrates frequently arise in Type V-K CASTs because they lack the TnsA endonuclease^45,46^. Using Pacbio SMRT long-read sequencing, we demonstrated that the wild-type VchCAST system predominantly generates simple insertions, whereas a D90A mutation in the TnsA active site favors cointegrates (>95%)^46^. Newly engineered Type V-K CAST systems (HELIX), that include a fused nicking homing endonuclease to mimic the function of TnsA, exhibit greatly increased simple insertion purity^45^, underscoring the key role of second-strand cleavage during donor DNA excision (Box 1).

In summary, CAST systems hold great value in genetically engineering both model and nonmodel bacteria, as well as in culture-independent editing of microbial communities. Microorganisms exist in dynamic and complex communities that synergistically interact through individual metabolic pathways and intermicrobial communication networks. Yet our understanding of these interactions is sparse, due in part to the limited availability of genetic tools to determine gene functions, which are often only applicable to pure cultures where these complex interactions cannot occur. One key example of a microbiome that can benefit from CAST engineering is that of the human gastrointestinal tract, which comprises a complex and diverse microbial community whose composition and spatial architecture are increasingly being appreciated as critical drivers of human health and behavior^78^. The ability to study and directly manipulate complex microbial communities in vivo, such as the gut microbiome, is critical for mechanistic studies of these microbial interactions and the development of novel therapeutics; however, the tools currently available remain severely limited and insufficient. For example, high-throughput sequencing offers only observational information, germ-free mammalian systems poorly reflect natural host–microbiome interactions, and probiotics suffer from limited temporal persistence in non-native environments. In the future, addressing these key limitations will require the development of new platforms for precision microbiome engineering, potentially combining programmable CASTs with broadly transmissible vectors for culture-independent microbial manipulation. These approaches would use CASTs not only to introduce edits, but also to stably embed desired genetic payloads and allow for long-term persistence in microbiomes. These future thrusts in microbial engineering will define a new paradigm for genetic studies of microbiomes from diverse environments (i.e., gut, soil, ocean, extremes), enabling scientists to harness the power of microorganisms for the benefit of human advancement. Lastly, the use of CASTs in eukaryotic organisms, and especially mammalian cells, holds enormous potential for future genome engineering applications related to human disease. In fact, recent advances by our group and others have applied both Type I-F^79^ and Type-V-K^45^ CAST systems to perform targeted DNA integration in human cells. Conventional nuclease-based genome engineering approaches, such as CRISPR–Cas9, create double-strand breaks that lead to undesirable and heterogeneous byproducts. CASTs offer substantial potential in eukaryotic cells, enabling single-step, RNA-guided integration of large payloads that can be effectively used to study or treat human genetic diseases. However, further work will be required to increase integration efficiencies, demonstrate on-target specificity, and relax the requirement for additional host factors^76,79^.

### Experimental design

**Fig. 4 |:**
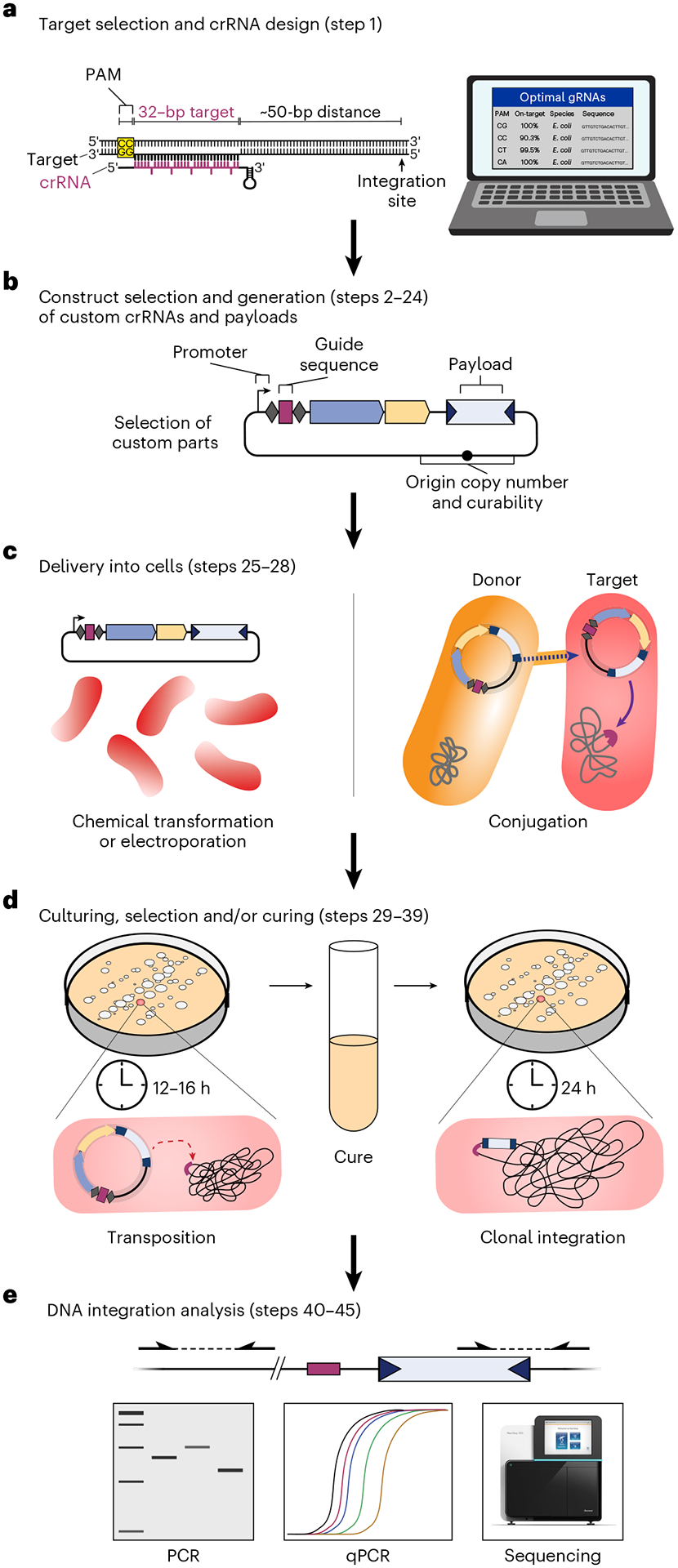
General CAST engineering workflow. **a**, The protocol workflow begins with the computational analysis of the bacterial genome(s) of interest to identify a 32-bp target site flanked by an appropriate 5′-CN-3′ PAM (in yellow), which minimizes the likelihood of off-target insertions. **b**, Appropriate vector(s) for the desired experiments are selected, and custom crRNAs and DNA payloads (and optional promoters) are cloned. **c**, The finalized vector is delivered into target bacteria using either chemical transformation, electroporation or conjugation. **d**, Target bacteria are incubated for 12–18 h on the appropriate selectable marker to allow efficient transposition to occur, followed by optional curing of the expression plasmid. **e**, RNA-guided DNA integration can be assessed using PCR and/or qPCR approaches. High-throughput sequencing may be used to systematically evaluate genome-wide specificity during the editing experiment.

The generation of programmable genomic insertions using CAST systems involves five main stages (Fig. 4): (1) designing the crRNA and target DNA sequence (Step 1), (2) cloning the crRNA guide sequence and custom genetic payload into appropriate vectors (Steps 2–24), (3) delivering one or more vector construct(s) into target cells (Steps 25–28), (4) culturing and selection (Steps 29–39), and (5) analyzing integration events (Steps 40–45), with optional isolation of desired clones. In the sections below, we provide detailed guidelines for each of these stages to enable use of CAST systems for different engineering scenarios and applications.

#### Target selection and crRNA design

The general workflow for target selection and crRNA design begins with selecting the desired genomic site for integration of the genetic payload, followed by identifying a 32-bp target sequence located ~50 bp away. Most insertions occur in a T-RL orientation, with the transposon right end integrated proximal to the target site (Fig. 1d), such that insertions in a preferred orientation can be generated by selecting a candidate target site either downstream or upstream of the integration site. The target sequence must be directly flanked by a compatible PAM recognized by QCascade, which is 5′-CN-3′ for most Type I-F CAST systems^33,41,80^. Off-target transposon insertions can occur when genomic sites are highly similar to the intended target site^32^. Therefore, the target sequence should be carefully selected such that guide RNAs with highly similar, partially matching target sites elsewhere in the genome are avoided. Mismatches within the seed region (positions 1–8) and the PAM distal region (positions 25–32) are discriminated particularly well by QCascade, whereas mismatches within positions 9–24 are discriminated less well^33,51^, all factors that should be taken into account when evaluating the risk of off-target insertions. Users are encouraged to perform analyses at candidate off-target sites to confirm the lack of undesired insertions for an isolated clone, and/or perform unbiased off-target insertion profiling.

#### Construct selection and generation of custom crRNAs and payloads

A list of CAST constructs suitable for many engineering applications is provided through Addgene (Supplementary Table 1). The suggested list includes single-plasmid pSPIN constructs for the VchCAST Type I-F system either encoded on a pCDF backbone (for use in *E. coli*), on a pBBR1 broad-host vector^81^ (for use in *E. coli* and related Gram-negative bacteria), or on a temperature-sensitive pSC101* backbone (for simple plasmid curing in *E. coli* cells^32,82^). VchCAST can also be delivered to cells as two separate plasmids, in which a pEffector vector encodes the guide RNA and all protein components, and a pDonor vector encodes the mini-Tn payload. These compatible plasmids are provided on *E. coli*-specific vector backbones and can be used in lieu of pSPIN for applications involving large genetic payloads that may be difficult to clone and deliver on a single pSPIN vector (Fig. 3). For most of the vectors, we recommend the RNA and protein components are expressed from a strong constitutive promoter (i.e., J23119), although different promoters that are weaker, inducible and/or specific to other desired bacterial species can alternatively be used. Although the overall rates of self-targeting for CASTs are generally low, we re-engineered a pSPIN variant, termed pSPIN-R, which encodes the CRISPR array proximal to the mini-Tn to repress self-targeting through target immunity^32,53^. This construct effectively restricts self-targeting-based vector inactivation but does exhibit slightly lower integration efficiencies compared with pSPIN.

Beginning with pEffector or pSPIN entry plasmids, new crRNA spacer sequences are cloned by ligating hybridized oligonucleotide pairs (outlined in Steps 2–23 and Box 2). For multiplexed applications, multiple spacers along with intervening repeat sequences can similarly be cloned using several overlapping oligo pairs (Box 2). Custom mini-Tn payload sequences can be cloned into pDonor or pSPIN vectors through various simple cloning strategies, and we provide steps for Gibson assembly within this protocol (Step 24). We have achieved robust integration activity with transposons ranging from ~300 bp to ~10 kb in size^32^. Given that natural CRISPR-associated transposons can be >100 kb in length^34,43^, payloads much larger than 10 kb can in theory be mobilized. However, the full-size range has not yet been systematically investigated. In general, optimal integration efficiencies in *E. coli* under certain experimental conditions were observed with mini-Tn constructs spanning 500–1,000 bp in size, while larger and smaller transposons may result in decreased efficiencies.

A recent report described the presence of a promoter within non-essential regions of the VchCAST transposon right end^33,35^, which can lead to leaky expression of encoded payload genes. In addition, improvements in product purity and efficiency using transposon variants containing truncated right ends missing this region have been reported by our group^32,33^. In light of this observation, we encourage the use of modified VchCAST mini-Tn designs in which the right end is truncated to a final length of 57 bp. Similar leaky expression may also occur with other homologous Type I-F CAST systems, and recent work reports a more systematic investigation of the minimal left and right end sequence requirements during VchCAST transposition^76^.

#### Delivery into cells

Within the context of common *E. coli* laboratory strains, vector delivery can be successfully achieved by simple heat-shock transformation of chemically competent cells. For other strains and species, we suggest high-efficiency electroporation as the default strategy for cellular transformation, particularly for experiments involving large plasmids and/or the combined use of pEffector and pDonor vectors. Of note, even with a low transformation efficiency, the high integration efficiency with CAST systems enables straightforward isolation of clones containing the desired genomic insertion.

For species or strains where electroporation is inefficient or impractical, and especially within complex bacterial communities^32,37,83^, we encourage the use of conjugation as an alternative route for transformation^84^. Bacteria naturally exchange plasmids through conjugation as a highly effective mean to share genetic material. We and others have utilized bacterial conjugation to efficiently deliver plasmids into isolates as well as complex bacterial communities, demonstrating its viability for CAST-mediated genome engineering^32,37^. Due to its broad-range capability, we highly recommend the RP4 transfer system to conjugatively transfer DNA from *E. coli* to several bacterial species. In this protocol, we describe steps to generate and transform chemically and electrocompetent *E. coli*, as well as a protocol for transforming *E. coli* through conjugation.

#### Culturing, selection and/or curing

After transformation and recovery, cells are usually plated on solid media with appropriate antibiotic selection. While integration can also be performed within a liquid culture, we have observed that solid plating during transposition reduces potential competitive growth effects within a heterogeneous cell population^32^. As the efficiencies of integration are generally high, we normally select only for antibiotic resistance encoded on the vector backbone(s). It is noteworthy to mention that many of the mini-Tn variants in our entry vectors encode a promoter-less chloramphenicol resistance gene that we originally selected as an arbitrary payload construct. For scenarios where selection for integration events is desired, a drug marker expression cassette should be cloned into the transposon. However, as vector-based expression of the transposon marker can also occur under conditions where the vector is stably maintained, users should perform selection only after curing cells of the plasmid. Alternatively, a ‘promoter capture’ approach can be used, whereby a transposon encoding a promoter-less marker gene is inserted downstream of an active genomic promoter. In this case, steps to ensure that there is no leaky vector-based expression, such as by truncating the transposon right end as discussed above, should be taken.

To allow transposition, *E. coli* cells are typically cultured at 37 °C over a period of ~18–24 h, unless the temperature-sensitive pSC101 vector is used. However, we have observed that longer incubation times at either 30 °C or 25 °C can strongly enhance integration efficiencies with VchCAST in *E. coli*, particularly for large DNA payloads^32^. This effect is not universal across other homologous Type I-F CAST systems, nor has it been tested in bacteria other than *E. coli*. However, different incubation temperatures can be easily tested in parallel while optimizing the system for a new target species, particularly for species that do not grow optimally at 37 °C. Multiple cycles of solid-medium culturing has also been shown to induce higher efficiencies for multiplexed integrations^32^, and dilution of cultures at early log phase may also enhance efficiencies when performing integration assays in liquid medium^32^.

If using pSPIN constructs on the temperature-sensitive pSC101 vector, cells can be cured of the plasmid after integration via liquid-medium growth at 37 °C in the absence of drug selection. Other backbones, such as pBBR1, can also be cured by culturing cells over several generations without drug selection, followed by frequent phenotyping on a selective medium, although with less robust results. While not described in this protocol, users may also explore other published methods for plasmid curing, such as the Cas9-based pCutamp system^59^.

#### DNA integration analysis

**Fig. 5 |:**
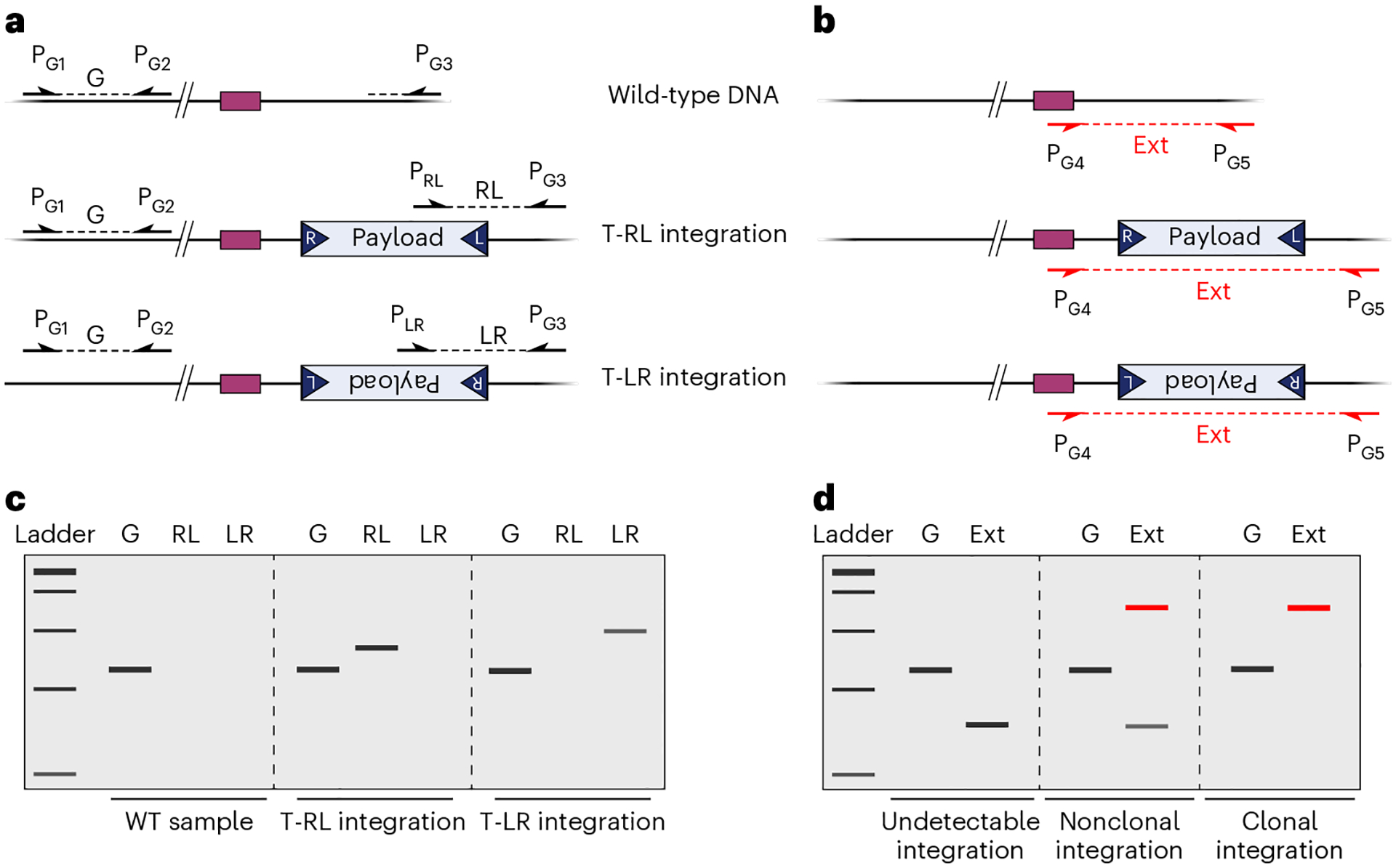
PCR and qPCR analysis of integration products. **a**, A schematic representation of junction primer pairs used for the analysis of integration events for unmodified, wild-type DNA (top), T-RL integration products (middle) and T-LR integration products (bottom). PCR product G, amplified by primer pair P_G1_ and P_G2_, refers to a reference gene that is unaffected by CAST targeting and serves as a benchmark for PCR and qPCR measurements. Integration products are detected by priming off the transposon and a genomic site downstream of the integration site, resulting in either junction PCR product RL (primer pair P_RL_ and P_G3_) or junction PCR product LR (P_LR_ and P_G3_). **b,** Integration products can also be detected using primers that bind upstream and downstream of the integration site, rather than the junction strategy shown in **a**. This method will produce PCR products that extend across the entire locus, whether unmodified or modified by CAST, using primers that are external (Ext) to the payload insertion. **c**, A schematic representation of a gel with the simulated PCR products from **a**. Note that individual colonies may also show bands indicative of both the T-RL and T-LR products, due to the presence of heterogeneous alleles present within single (nonclonal) colonies. **d**, A schematic representation of a gel with the simulated external PCR products from **b**. The primers (P_G4_ and P_G5_) will generate a PCR product for both the unmodified (WT) DNA and both integration products, with the product size identifying the presence of the desired edit. Note that this method is less sensitive than junction PCR, and that colonies may yield bands for both potential products (unmodified and integrated) due to nonclonality.

Transposon insertions at the target site can be routinely detected by genotyping using targeted PCR^32,33^. A standard PCR strategy probes for the existence of genome–transposon junctions at the target locus, using genome-specific and transposon-specific primer pairs (Fig. 5a). Since the VchCAST system produces a low level of T-LR insertions, multiple primers can be designed to probe for both possible orientations, if desired. To evaluate the efficiency of integration, qPCR can be used to quantify the proportion of gDNA molecules containing the newly formed junctions^32,33^. In this protocol, we also describe a simple PCR strategy involving two genome-specific primers flanking the transposon insertion, which is useful for isolating clonal integrants (Fig. 5b).

Importantly, we have consistently observed that *E. coli* colonies are often genetically heterogeneous and nonclonal after a single night of culturing and drug selection^33^. Diagnostic PCR analyses demonstrate the concurrent presence of all possible products (i.e., no integration, T-RL integration and T-LR integration), indicating that transposon insertion is slower than the cell doubling time and thus multiple alleles are propagated within the same colony. Therefore, when isolating clonal integrants, we advise to replate cells at least once, to allow integration products to be homogeneously fixed within the population of cells in the colony. In addition, we recommend estimating population-wide CAST on-target integration events via PCR/qPCR (Step 45). While beyond the scope of this protocol, it may be worthwhile to estimate off-target insertion events performing Tn-seq, a technique previously described in our work^32^. We encourage users to perform population-wide analysis to troubleshoot new crRNAs, variant CAST systems or in new target bacterial species.

## Materials

### Biological materials

- *E. coli* chemically competent strains for cloning:
  - NEB Turbo (NEB, cat. no. C2984)
  - NEB 10-beta (NEB, cat. no. C3019)
  - NEB Stable (NEB, cat. no. C3040)
- Target bacterial strain-of-interest:
  - E.g., *E. coli* BL21(DE3) chemically competent cells (Sigma cat. no. CMC0014)
  - E.g., *E. coli* BL21(DE3) electrocompetent Cells (Sigma cat. no. CMC0016)
  - E.g., *E. coli* MG1655 (ATCC, cat. no. 700926)
- Conjugative donor strain *EcGT2* (ATCC, cat. no. 47055)

### Reagents

- Addgene plasmid list (Supplementary Table 1)

#### Common reagents

- Spectinomycin dihydrochloride pentahydrate (Gold Biotechnology, cat. no. S-140–5)
- Kanamycin monosulfate (Gold Biotechnology, cat. no. K-120–5)
  ▲**CAuTIoN** May cause infertility and damage the unborn child. Wear protective clothing, gloves, face and eye protection.
- Carbenicillin disodium (Gold Biotechnology, cat. no. C-103–5)
  ▲**CAuTIoN** May cause an allergic skin reaction and, if inhaled, asthma symptoms or breathing difficulties. Wear gloves and eye and face protection. In case of inadequate ventilation wear respiratory protection.
- LB medium (Thermo Fisher Scientific, cat. no. BP9723–2)
- LB agar medium (Thermo Fisher Scientific, cat. no. BP9724–500)
- Agarose, low electroendosmosis (EEO), molecular biology grade (Fisher Scientific, cat. no. BP160–500)
- TAE buffer, 50× (Bio-Rad, cat. no. 1610773)
- Gel loading dye, 6× (included with enzyme, or purchased separately from NEB, cat. no. B7024S)
- SYBR Safe DNA gel stain (Thermo Fisher Scientific, cat. no. S33102)
- 1 kb DNA ladder (Gold Biotechnology, cat. no. D010–500)
- 100 bp Plus DNA ladder (Gold Biotechnology, cat. no. D003–500)
- Milli-Q (MQ) water
- Liquid nitrogen
  ▲**CAuTIoN** Handle with care to prevent freeze burns.
- Absolute ethanol (Fisher Scientific, cat. no. BP2818100)
  ▲**CAuTIoN** Highly inflammable compound. Harmful if swallowed, it may cause serious eye irritation and organ damage. Keep away from heat and wear appropriate protection.
- Isopropanol (Fisher Scientific, cat. no. A426P-4)

#### Competent cell preparation

- Glycerol (Sigma, cat. no. G5516)
- Dimethyl sulfoxide (DMSO) (Sigma, cat. no. D2650)
  ▲**CAuTIoN** Contains impurities that can cause health issues. It rapidly absorbs through the skin. In case of contact rinse with plenty of water. Use appropriate eye protection and gloves as well as respiratory protection for vapors of organic compounds.
- MgCl_2_ hexahydrate powder (Sigma, cat. no. M2670)
- CaCl_2_ dihydrate powder (Sigma, cat. no. 223506)

#### Conjugation

- Square plates (Thermo, cat. no. 242811)
- Six-well plates (Fisher Scientific, cat. no. 08-772-49)
- 1× PBS pH 7.4 (Gibco, cat. no. 10010023, or home-made)

#### DNA extraction kits

- QIAprep Spin Miniprep Kit (Qiagen, cat. no. 27115)
- QIAquick Gel Extraction Kit (Qiagen, cat. no. 28706×4)
- MinElute Gel Extraction Kit (Qiagen, cat. no. 28604)
- Wizard Genomic DNA Purification Kit (Promega, cat. no. A1125)

#### PCR and qPCR

- Q5 Hot Start High-Fidelity DNA Polymerase (NEB, cat. no. M0493S/L)
- dNTP mix, 10 mM (NEB, cat. no. N0447S/L)
- Q5 reaction buffer, 5× (included with enzyme, or purchased separately from NEB, cat. no. B9027S)
- (Optional) OneTaq Quick-Load 2× Master Mix (NEB, cat. no. M0486S/L), for genotyping only
- Oligonucleotide primers for PCR, from IDT or preferred vendor
- SsoAdvanced Universal SYBR Green Supermix (Bio-Rad, cat. no. 1725270–1725275)

#### Construct cloning

- T4 DNA Ligase (NEB, cat. no. M0202S/T/L/M)
- T4 Polynucleotide Kinase (NEB, cat. no. M0201S/L)
- NEBuilder HiFi DNA Assembly Master Mix (NEB, cat. no. E2621S/L/X)
- BsaI–HFv2 restriction enzyme (NEB, cat. no. R3733S/L)
- BamHI–HF restriction enzyme (NEB, cat. no. R3136S/L/T/M)
- HindIII–HF restriction enzyme (NEB, cat. no. R3104T/M)
- KpnI–HF restriction enzyme (NEB, cat. no. R3142S/L/M)
- PstI–HF restriction enzyme (NEB, cat. no. R3140S/L/T/M)
- XhoI restriction enzyme (NEB, cat. no. R0146S/M)
- Bsu36I restriction enzyme (NEB, cat. no. R0524S/L)
- SalI restriction enzyme (NEB, cat. no. R0138S/T/L/M)
- CutSmart or rCutSmart buffer, 5× (included with enzyme, or purchased separately from NEB, cat. no. B7204S/B6004S)
- T4 DNA Ligase reaction buffer, 10× (included with enzyme, or purchased separately from NEB, cat. no. B0202S)

### Equipment

#### Glassware

- Electroporation cuvettes, 1 or 2 mm gap (Fisher, cat. no. P41050)
- Sterile glass plating beads (VWR, cat. no. 76005–122)
- Assorted glass bottles (Thermo, cat. no. 045900)

#### Plasticware

- Eight-strip PCR tubes with attached flat caps, 0.2 mL (Sigma, cat. no. BR781332)
- Conical polypropylene centrifuge tubes, 50 mL (Fisher, cat. no. 14-959-49A)
- Two-sided disposable polystyrene plastic cuvettes, 1.5–2 mL (VWR, cat. no. 97000-590)
- Microcentrifuge tubes, 1.7 mL (Sigma, cat. no. CLS3620–500)
- Sterile 100 × 15 mm Petri dishes
- Sterile plastic inoculation loop
- Hard-Shell 384-well qPCR plates, clear shell/white wells
- Microseal ‘B’ qPCR plate sealing film
- Serological pipettes, 2–25 mL
- Sterile baffled plastic Erlenmeyer flask, 250 mL or 2 L
- Vacuum filter/storage bottle system, 0.22 μm pore 33.2 cm^2^ polyethersulfone membrane, sterile (Corning, cat. no. 431097)

#### Tools and instruments

- Static incubator (Fisher, cat. no. 15-103-0516)
- Shaking incubator (VWR, cat. no. 76628-592)
- Tabletop centrifuge (Eppendorf, cat. no. 022625501)
- Benchtop microcentrifuge (Eppendorf cat. no. 5429000133)
- Microvolume spectrophotometer (Fisher, cat. no. ND2000CLAPTOP)
- 96-Well thermocycler (Thermo, cat. no. 4375305)
- CFX384 384-well qPCR system (Bio-Rad, cat. no. 1845097)
- Gel electrophoresis power supply (Bio-Rad, cat. no. 1645070)
- Gel electrophoresis tank with casting trays (Bio-Rad, cat. no. 1704402)
- Gel imaging system (Bio-Rad, cat. no. 12009077)
- GenePulser bacterial electroporation system (Bio-Rad, cat. no. 1652660)
- Heat block for microcentrifuge tubes (Fisher, 88-870-006)
- Cell culture spectrophotometer (Thomas Scientific, cat. no. 23A00C979)
- Benchtop vortex (Fisher, cat. no. 02-215-414)
- Blue-light gel platform (Thermo Fisher, cat. no. G6600)
- Metal razors (Fisher, cat. no. 12–640)
  ▲**CAuTIoN** Sharp item that can lacerate the skin. Take appropriate precautions against sharp injury and immediately dispose in a sharp container after use.
- Pipets: p1000, p200, p20, p2 (Rainin. cat. no. 8479899599)
- Sterile pipette tips (Rainin. cat. nos. 30389271, 30389276, 30389270)

#### Software

- Benchling (https://www.benchling.com/), or preferred comparable software
- CAST crRNA design tool (https://github.com/sternberglab/CAST-guide-RNA-tool)

### Reagent setup

#### 50% (vol/vol) glycerol solution

Mix 200 mL of glycerol with 200 mL of MQ water. Sterilize by autoclaving. Cool down on ice before use. The solution can be stored at 4 °C for <6 months.

#### 10% (vol/vol) glycerol solution

Mix 50 mL of glycerol with 450 mL of MQ water. Sterilize by autoclaving or sterile filtration. Cool down on ice before use. Store at 4 °C for < 6 months.

#### 1 M MgCl_2_ solution

Dissolve 101.65 g of MgCl_2_ hexahydrate powder in 250 mL of MQ water. Once dissolved, adjust the volume to 500 mL, using MQ water. Sterilize by autoclaving or sterile filtration. Store at room temperature (22–23 °C) for 1 year.

#### 1 M CaCl_2_ solution

Dissolve 73.51 g of CaCl_2_ dihydrate powder with MQ water. Once dissolved, adjust the volume to 500 mL, using MQ water. Sterilize by autoclaving or sterile filtration. Store at room temperature for 1 year.

#### 100 mg/mL spectinomycin solution (1,000× stock)

Dissolve 5.0 g of spectinomycin powder with MQ water to reach a final volume of 50 mL, and sterile filter. Aliquot the solution into microcentrifuge tubes and store at −20 °C for <12 months.

#### 50 mg/mL kanamycin solution (1,000× stock)

Dissolve 2.5 g of kanamycin powder with MQ water to reach a final volume of 50 mL, and sterile filter. Aliquot the solution into microcentrifuge tubes and store at −20 °C for < 12 months.

#### 50 mg/mL carbenicillin solution (1,000× stock)

Dissolve 2.5 g of carbenicillin powder with MQ water to reach a final volume of 50 mL, and sterile filter. Aliquot the solution into microcentrifuge tubes and store at −20 °C for <12 months. Ampicillin may also be used in lieu of carbenicillin.

#### 50 mg/mL diaminopimelic acid (DAP) solution (1,000× stock)

Dissolve 2.5 g of DAPI powder with MQ water to reach a final volume of 50 mL, and sterile filter. Aliquot the solution into microcentrifuge tubes and store at −20 °C for <12 months.

## Procedure

### Part 1: target selection and crRNA design

● TIMING 3 h

### Construct selection and crRNA design

● TIMING 3 h

1. Select the appropriate CAST vector construct(s) and design crRNA oligos using the crRNA design tools, as detailed in Box 2.
  ▲**CRITICAL STEP** Chose an appropriate CAST vector construct or combinations of constructs (refer to the ‘Experimental design’ section for further details). Generally, we recommend using the VchCAST (Tn*6677*) constructs, specifically the pSC101*–pSPIN vector for *E. coli* applications that require efficient plasmid curing, or the pBBR1–pSPIN vector for integration experiments in *E. coli*, *Klebsiella*, *Pseudomonas* or related Gram-negative bacteria.

### Part 2: generation of custom crRNAs and payloads

● TIMING 2 d

#### Cloning custom crRNA spacers

● TIMING 2 d

- Prepare the reaction mix to digest pEffector or pSPIN plasmids, following the instructions provided below.

**Table:** 

Component	Amount (μL)	Final concentration
Purified pEffector or pSPIN plasmid	Variable (see below)	
10× Cutsmart buffer	5	1×
BsaI-HFv2 (NEB)	2	
MQ water	Up to 50 μL	
Total	50
  Suggested amounts of input DNA:

**Table:** 

Component	Amount (μL, for ~150 ng/μL plasmid aliquots)	Final concentration
pCDF-pEffectors	17	2500 ng
pSC101*-pSPINs	33	5000 ng
pBBR1-pSPINs	20	3000 ng
pCDF-pSPINs	20	3000 ng
- Incubate the reaction at 37 °C for 2 h.
- Prepare a 1% agarose gel in 1× TAE buffer and supplement it with 1% SYBR Safe or another appropriate DNA staining reagent.
- Add 6× loading dye to each reaction tube.
- Run 50 μL of the digestion at 140 V for 30 min in 1× TAE buffer on a 1% agarose gel along with 10 μL of DNA ladder.
- Visualize the agarose gel on a UV transilluminator system and cut the band of interest (~12–14 kb in size, depending on plasmid construct) using a clean blade or razor.
- Extract the digested vector from the gel using the QIAquick Gel Extraction Kit (Qiagen) according to the manufacturer’s guidelines.
  ▲**CRITICAL STEP** If the digested band is faint, the MinElute Gel Extraction Kit (Qiagen) should be used instead to obtain sufficiently concentrated DNA.
- Elute the digested vector in 30 μL of elution buffer.
  ▲**CRITICAL** Use 10 μL of elution buffer if using the MinElute Gel Extraction Kit.
  ■**PAuSE PoINT** DNA can be stored for several months at −20 °C.
- During digestion/gel electrophoresis, prepare hybridized oligoduplex for ligation, by mixing 2.5 μL of each oligoduplex and 20 μL of MQ water, heating the mixture to 95 °C for 2 min and finally allowing to cool down to room temperature.
  ▲**CRITICAL STEP** Generally, primers are purchased from a vendor already containing 5′-phosphorylation modification. Alternatively, if performing 5′-phosphorylation in house, please follow the steps details in Box 3.
- Dilute the oligoduplex following option A if using a single oligoduplex and option B if ligating multiple (up to five) oligoduplexes.
  - Single oligoduplex:
    - If ligating a single oligoduplex (i.e., encoding a single spacer), prepare a 1:200 dilution (50 nM final concentration) of the oligoduplex mixture in MQ water and use it in a ligation reaction as outlined in the first table in Step 12.
  - Multiple oligoduplexes:
    - If ligating multiple oligoduplexes (i.e., encoding multiple spacers; Supplementary Table 2), prepare a 1:50 dilution (50 nM final concentration) of each oligoduplex mixture in MQ water, and use them in a ligation reaction, as outlined in the second table in Step 12.
- Set up the ligation reaction on ice, as outlined in the tables below, and add the T4 ligase last, to prevent high levels of spurious intramolecular ligation of the digested vector. We suggest a no-template ligation reaction as a negative control to estimate the background frequency of template religation.

**Table:** 

Component	Amount (μL)	Final concentration
50 nM hybridized/phosphorylated oligoduplex	2	10 nM
~25 ng/μL digested/purified plasmid	2	50 ng
T4 DNA Ligase (NEB)	0.5	
10× T4 DNA Ligase buffer (NEB)	1	1×
MQ water (up to 10 μL)	4.5	

Component	Amount (μL)	Final concentration
Each 50 nM hybridized/phosphorylated oligoduplex	Variable	10 nM
~25 ng/μL digested/purified plasmid	2	50 ng
T4 DNA Ligase (NEB)	2.5	
10× T4 DNA Ligase buffer (NEB)	5	1×
MQ water	Up to 50 μL
- Incubate the ligation reaction at room temperature for 30 min. Mix 10 μL of the ligation reaction with 50 μL of chemically competent *E. coli* cells (cloning strain) on ice.
  ▲**CRITICAL STEP** The use of commercially available, chemically competent *E. coli* (NEB 10-beta, NEB Turbo, NEB Stable) is recommended.
- Incubate on ice for 15 min, heat shock at 42 °C for 30 s, then place back on ice for 5 min.
- Transfer the entire transformation mix to a microcentrifuge tube containing 950 μL LB media. Recover with shaking (120 rpm) at 37 °C for 1 h.
  ▲**CRITICAL STEP** If cloning into pSC101* plasmids, recover at 30 °C for 2 h.
- Plate 100 μL of the transformed bacteria on an LB agar plate containing the appropriate antibiotic selection (Supplementary Table 1).
- Pellet the remaining 900 μL of the transformed bacteria by centrifuging the tube at 4,000*g* for 5 min.
- Discard the supernatant and resuspend the pellet by pipetting in 100 μL of LB medium.
- Plate the cells on a LB agar plate containing the appropriate antibiotic selection and spread evenly.
- Once all visible liquid has dried off from the plates, incubate the plates at 37 °C overnight until colonies are visible.
  ▲**CRITICAL STEP** If cloning into pSC101* plasmids, incubate cells at 30 °C overnight. Plates may take up to 24 h to produce visible colonies.
  ■**PAuSE PoINT** Plates can be stored for a month at 4 °C.
  ◆ TROUBLESHOOTING
- In a 50 mL conical tube, inoculate three to six colonies from the plates in Step 19 in 5 mL LB medium containing 1× concentration of the appropriate antibiotic. Incubate overnight with shaking (120 rpm) at 37 °C.
  ▲**CRITICAL STEP** If cloning into pSC101* plasmids, incubate cells at 30 °C overnight.
- (Optional) For long-term storage of the cell culture at −80 °C, prepare a glycerol stock by mixing 300–500 μL of turbid overnight culture with an equal volume of sterile 50% glycerol. If more plasmid is needed, inoculate LB media as above using a small scraping of this frozen stock; alternatively, transform a cloning *E. coli* strain with 5–10 ng of miniprepped plasmid and inoculate LB media using colonies retrieved from this transformation.
- Extract plasmid DNA from each culture using QIAprep Miniprep Kit or similar plasmid extraction kit, and verify the CRISPR array sequence with Sanger sequencing, using the list of primers suggested in Supplementary Table 2.
  ▲**CRITICAL STEP** We strongly encourage the use of whole-plasmid sequencing services such as Plasmidsaurus or Primordium to verify integrity of the entire vector.

#### (optional) Cloning custom transposon DNA

● TIMING 2 d

- (Optional) Perform molecular cloning to replace the default payload sequence with a custom, user-desired payload in VchCAST (option A) or for altering VchCAST transposon ends to increase integration efficiency (option B). These steps are optional for users that require custom payloads that are different from the promoter-less chloramphenicol payloads in the plasmids we provide.
  ▲**CRITICAL STEP** We routinely replace payloads depending on the desired experimental outcome and recommend tailoring the payload to the experiment. These steps can be followed before performing the crRNA spacer cloning (Steps 2–21), depending on the desired downstream application (e.g., inserting the same custom transposon at multiple different target sites). Moreover, we have recently discovered that altering the transposon ends can improve the system for various applications, including the generation of functional linkers^76^. We advise users to follow the example provided in option B as a guideline for other types of changes in the transposon ends, such as truncations and mutations.
  - Cloning custom transposon payload sequence in VchCAST
    - Set up the digest reaction for either pDonor or pSPIN vectors following the instruction provided below:

**Table:** 

Component	Amount (μL)	Final concentration
Purified pEffector or pSPIN plasmid	Variable (see below)	
10× Cutsmart buffer	5	1×
XhoI (Supplementary Table 3)	1	
PstI (Supplementary Table 3)	1	
MQ water	Up to 50 μL
      Suggested amounts of input DNA:

**Table:** 

Plasmid	Amount (μL, for ~150 ng/μL plasmid aliquots)	Final concentration (ng)
pUC19 pDonor	17	2,500
pSC101* pSPINs	33	5,000
pBBR1 pSPINs	20	3,000
pCDF pSPINs	20	3,000
    - Incubate at 37 °C for 2 h.
    - Perform gel electrophoresis and extraction of the band of interest (~11–13 kb for pSPINs, and ~2.7 kb for pDonor vector) from the gel as described in Steps 4–9.
    - Amplify custom payload DNA by PCR for Gibson cloning using Q5 Hot Start Polymerase (NEB) as detailed below. Alternatively, a Q5 Hot Start 2× Mastermix or similar high-fidelity polymerases can also be used according to manufacturer’s guidelines.

**Table:** 

Component	Amount (μL)	Final concentration
Template DNA	1	
Custom primer 1, 10 μM (see Supplementary Table 2)	2.5	0.5 μM
Custom primer 2, 10 μM (see Supplementary Table 2)	2.5	0.5 μM
dNTPs, 10 mM	1	0.2 mM
Q5 Hot Start DNA Polymerase (NEB)	0.5	
5× Q5 Reaction Buffer	10	1×
MQ water (up to 50 μL)	32.5
      ▲**CRITICAL STEP** Plasmid-derived template DNA should be diluted to <10 ng/μL; for amplifying from gDNA of bacteria, users should try a range of dilutions until a clean amplification is achieved.
    - Perform PCR in a thermocycler using the following cycling parameters:

**Table:** 

Cycle number	Denature	Anneal	Extend	Hold
1	98 °C, 30 s	–	–	–
2–30	98 °C, 10 s	X °C, 20 s	72 °C, 30 s/kb	–
31	–	–	72 °C, 2 min	–
32	–	–	–	4 °C
      ▲**CRITICAL STEP** Vary the temperature of the annealing step according to primer binding sites and the polymerase used. The annealing temperature can be estimated using the online calculator available on the NEB website (https://tmcalculator.neb.com).
    - Confirm the successful amplification of the desired PCR product by performing gel electrophoresis and visualizing the gel using a UV transilluminator, followed by the purification of the DNA from the gel as described in Steps 4–9.
    - In a PCR strip tube, assemble the Gibson assembly reaction as detailed below.

**Table:** 

Component	Amount (μL)	Final concentration
Purified digested plasmid (~25 ng/μL)	3	75 ng
Purified PCR insert (~50 ng/μL)	3	150 ng
NEBuilder HiFi Assembly 2× Mix	6	1×
Total	12
      ▲**CRITICAL STEP** For large multikilobase PCR inserts, increase the amount of purified PCR product (80–100 ng) in Gibson reaction.
    - Incubate the reaction at 50 °C for 1–2 h in a thermocycler.
    - Transform competent *E. coli* cells with the entire Gibson reaction.
    - Recover, plate, and inoculate colonies as detailed in Steps 13–21.
      ◆TROUBLESHOOTING
    - Confirm the transposon payload sequence by plasmid sequencing. (i.e., sanger, Plasmidsaurus).
  - Alter transposon ends via truncation of the VchCAST transposon right end to a 57-bp sequence.
    - Digest pDonor or pSPIN setting up the the following reaction mix.

**Table:** 

Component	Amount (μL)	Final concentration
Purified pEffector or pSPIN plasmid	Variable (see below)	
10× Cutsmart buffer	5	1×
Enzyme A (Supplementary Table 3)	1	
Xhol (NEB)	1	
MQ water	Up to 50 μL
      Digestion enzymes and suggested input DNA amounts:

**Table:** 

Plasmid	Amount (μL, for ~150 ng/μL plasmid aliquots)	Final concentration (ng)
pUC19 pDonor	17	2,500
pSC101* pSPINs	33	5,000
pBBR1 pSPINs	20	3,000
pCDF pSPINs	20	3,000
    - Incubate the reaction at at 37 °C for 2 h.
    - Perform gel electrophoresis and extraction of the digested plasmid (expected band sizes are ~12–14 kbp for pSPINs, and ~3.3 kbp for pDonor) as described in Steps 4–9.
    - Generate phosphorylated hybridized oligoduplexes separately for primer pairs (VchCAST_RE_fw1/VchCAST_RE_rv1) and (VchCAST_RE_fw2/VchCAST _RE_rv2) (Supplementary Table 2) as described in Box 3. Dilute each oligo duplex 1:50 in MQ water and set up the ligation reaction as detailed below.

**Table:** 

Component	Volume (μL)
Each 1:50 diluted oligoduplex	1.5
Purified plasmid digest (~25 ng/μL)	2
T4 DNA Ligase (NEB)	0.5
10× T4 DNA Ligase buffer (NEB)	1
MQ water (up to 10 μL)	3.5
      ▲**CRITICAL STEP** Prepare the ligation reaction on ice and add the T4 ligase enzyme last to prevent high levels of spurious ligation of the digested vector.
    - Incubate the ligation reaction at room temperature for 30 min.
    - Transform cells with entire ligation reaction, recover, plate and inoculate colonies as described in Steps 13–21.
      ◆TROUBLESHOOTING
    - Confirm transposon right end sequence by Sanger sequencing (the suggested primer is in Supplementary Table 2).

### Part 3: delivery into cells

● TIMING 3–4 d

#### Perform transposition in target strain

● TIMING 3–4 d

- Transform competent cells with pSPIN constructs either by following the steps in option A for chemical transformation of common *E. coli* strains, or by electroporation option B if targeting other strains or species. Alternatively, perform transposition following the Steps for bacteria conjugation (option C), which is especially useful for delivery into strains that are recalcitrant to transformation.
  ▲**CRITICAL STEP** If using a pEffector–pDonor combination, electroporation (option B) is recommended to achieve sufficient transformation efficiency; however, chemical transformation can still be used, especially when using highly competent commercial *E. coli* cells. Transformation of two plasmids at the same time is usually less efficiency than transforming one plasmid. To ameliorate transformation efficiency, cells can be first transformed with either pEffector or pDonor, followed by generation of chemically competent cells (as in Box 4), and then these competent cells harboring the first plasmid can be transformed with the second plasmid.
  ▲**CRITICAL STEP** We encourage users to perform in parallel a transformation using the respective nontargeting entry version of the vector constructs (meaning the vector before cloning of the desired custom crRNA spacer sequence). This is an important negative control to test for non-CAST-mediated integration and it should result in no integration events at the target site.
  ◆TROUBLESHOOTING
  - **Delivery of DNA constructs by chemical transformation**
    - On ice, mix 200–300 ng of each plasmid with 50 μL of chemically BL21 competent cells. If performing a two-plasmid transformation, use 300–400 ng of each construct.
    - Incubate on ice for 15 min, heat shock at 42 °C for 30 s, then return the tubes on ice for 5 min.
    - Add each transformation to a microcentrifuge tube containing 950 μL of LB media.
    - Recover with shaking (120 rpm) at 37 °C for 1–2 h.
      ▲**CRITICAL STEP** Recover at 30 °C for 2 h if using pSC101* constructs.
    - Plate 100 μL of the cell suspension on a LB agar plate containing the appropriate antibiotic selection (Supplementary Table 1).
    - Pellet the remaining 900 μL of the cell suspension, discard the supernatant and resuspend the pellet by pipetting in 100 μL LB medium before plating the whole sample on a LB agar plate supplemented with the right antibiotic selection.
    - Ensure that all visible liquid pools have dried off from plates before incubating the plates at 37 °C overnight.
      ▲**CRITICAL STEP** Plates can alternatively be incubated at 30 °C for 28–30 h, or at 25 °C for 40–48 h, which may induce higher integration efficiencies. We recommend incubating the plates at 30 °C or lower if using pSC101* constructs.
  - Delivery of DNA construct by electroporation
    - Prechill 1 mm electroporation cuvettes on ice. Meanwhile, prewarm 950 μL of LB medium in microcentrifuge tubes at 37 °C using a heat block or an incubator.
    - On ice, mix 50–100 ng of each plasmid with 40 μL of BL21 electro-competent cells. If performing a two-plasmid transformation, use 100–200 ng of each plasmid.
    - Pipette the mixture prepared in Step 25b(ii) into a cold electroporation cuvette.
    - Electroporate with a GenePulser electroporator at 2,000 kV, 25 μF and 200 Ohms. Arc times in the range of 4.5–5.1 ms are considered a highly efficient electroporation.
      ▲**CRITICAL STEP** Immediately pipette 700 μL of prewarmed LB medium, mix by pipetting and transfer the entire mixture back into the tube. Delays in adding recovery medium to electroporated cells can reduce the transformation efficiency.
    - Recover cells with shaking (120 rpm) at 37 °C.
      ▲**CRITICAL STEP** Recover at 30 °C for 2 h if using pSC101* constructs.
    - Plate the cells on a LB agar plate containing the right antibiotic selection and grow overnight at 37 °C.
      ▲**CRITICAL STEP** Plates can alternatively be incubated at 30 °C for 28–30 h, or at 25 °C for 38–44 h, which may induce higher integration efficiency. Incubate plates at 30 °C or lower if using pSC101* constructs.
  - Bacterial conjugation
    ▲**CRITICAL** A conjugative method for delivery is only viable with pSPIN constructs that are engineered with an origin of transfer, a necessary component for conjugation, and delivered with a conjugative strain that harbors genetically encoded RP4 conjugative machinery. The delivery method is reliant on a donor strain transformed with pSPIN. A commonly used donor strain is *E. coli* EcGT2, which utilizes RK2-based conjugal transfer^85^. This *EcGT2* donor strain is auxotrophic for the essential cell-wall component DAP, thus requiring DAP supplementation (50 μg/mL) in the growth media. This allows for counterselection of the donor after conjugation.
    ▲**CRITICAL** We recommend performing in parallel a transformation using the respective nontargeting entry version of constructs (before custom crRNA spacer cloning) to use as a negative control.
    - Generate chemically competent EcGT2 donor strain harboring pSPIN by chemical transformation following the procedure provided in Box 4.
    - Follow the procedure in Box 5 for the conjugative delivery of pSPIN from donor strain to recipient.
      ◆TROUBLESHOOTING
    - Select for positive transconjugants following the procedure in Box 6.

#### (optional) Estimation of conjugation efficiency

● TIMING 1 h

▲**CRITICAL** Using serial dilutions of conjugation reactions, processed in Box 6, the conjugation efficiency from donor to recipient can be estimated as detailed in the following steps by comparing colony counts among conjugation reactions, donor-only reactions and recipient-only reactions.

- After 12–24 h incubation of spots (Box 6), remove plates from incubator.
- Count the number of colonies for the two highest dilution series that grew on LB only and LB supplemented with the appropriate antibiotic selection for the following samples: conjugation reactions, donor-only reactions and recipient-only reactions.
- To calculate conjugation efficiency, apply the following calculation:
$$\frac{\frac{\left(R\times 10^{n}+R\times 10^{n-1}\right)}{2}}{\frac{\left(C\times 10^{n}+C\times 10^{n-1}\right)}{2}}$$
  where *R* is the number of c.f.u. in the selective plates and *C* is the number of c.f.u. in nonselective plates, at a given 10^n^ dilutions, where *n* is the fold dilution in the series of dilutions plated (i.e., 10^−3^).
  ▲**CRITICAL STEP** If LB plates were incubated without DAP supplementation, it is crucial that no growth be observed for donor-only reactions. If growth is observed, conjugation efficiency results will be inconclusive.
  ▲**CRITICAL STEP** Growth is expected for transconjugants and for recipient-only reactions on LB plates, but only growth of transconjugants is expected on LB supplemented with the appropriate antibiotic. If recipient-only reactions exhibit growth on the antibiotic, it is necessary to determine the minimum inhibitory concentration and repeat the experiment at that concentration of antibiotic.
  ▲**CRITICAL STEP** For the transconjugant, divide the number of colonies in the highest dilution on LB-only plates by the number of colonies in the highest dilution on LB supplemented with appropriate antibiotic. This final number is the conjugation efficiency.

### Part 4: culturing, selection and/or curing

● TIMING 3–4 d

#### Isolating clonal integrants by PCR

● TIMING 18 h

▲**CRITICAL** The steps below describe isolation of clonal integrants from initial transformations/conjugations in Step 25, achieved by genotyping random colonies using colony PCR. We want to emphasize that if the goal is clonal isolation of an integrant, additional passaging will be required (at least once) to homogenously fix the integration product, as colonies will typically not be clonal after a single transformation step. Depending on genomic insertion site or transposon payload genes, users may instead opt to isolate clonal integrants through phenotyping, then subsequently confirm insertions by PCR. For detection of clonal insertions, an external–external PCR strategy is used (Fig. 5c,d). We recommend performing an additional PCR reaction for an unmodified genomic locus (as in Fig. 5a), as a positive control that should yield a band for the PCR reaction.

▲**CRITICAL** For multiplexed experiments, selection for clonal integration can be performed by performing genotyping PCR at all target sites simultaneously. If colonies with clonal insertion at all sites are not found from the first round of PCR, colonies with clonal insertions at several sites can be restreaked for an additional overnight incubation, followed by further PCR genotyping.

- From single colonies derived from plates after Steps 25 or 28, pick 10–20 colonies and inoculate them in 40 μL of MQ water.
  ▲**CRITICAL STEP** Include one or two colonies from a negative control plate.
- Using a new LB agar plate supplemented with the appropriate antibiotic selection, spot 1 μL of each cell resuspension and incubate plate at 37 °C overnight once the spots have dried completely.
  ▲**CRITICAL STEP** Incubate plate at 30 °C if using pSC101* constructs.
- Heat the remaining cell resuspension at 95 °C for 10 min, and then cool to room temperature. Dilute each lysate 1:20 in 40 μL of MQ water.
- Set up a PCR reaction for each diluted lysate as detailed below.

**Table:** 

Component	Amount (μL)	Final concentration
Diluted lysates (Step 31)	5	
10 μM Primer 1 (Supplementary Table 2)	0.625	0.5 μM
10 μM Primer 2 (Supplementary Table 2)	0.625	0.5 μM
10 mM dNTPs	0.25	0.2 mM
Q5 Hot Start DNA Polymerase (NEB)	0.125	
5×Q5 Reaction Buffer	2.5	1×
MQ water (up to 12.5 μL)	3.375
  ▲**CRITICAL STEP** For genotyping, high-fidelity polymerases such as Q5 (NEB) are recommended. However, lower-fidelity polymerases such as OneTaq (NEB) or equivalent can also be used. Also, preparation of a batch mastermix is recommended to reduce pipetting errors. For primer design refer to Fig. 5c.
- Perform PCR in a thermocycler using the following cycling parameters:

**Table:** 

Cycle number	Denature	Anneal	Extend	Hold
1	98 °C, 30 s	–	–	–
2–30	98 °C, 10 s	*X* °C, 20 s	72 °C, 30 s	–
31	–	–	72 °C, 2 min	–
32	–	–	–	4 °C
  ▲**CRITICAL STEP** Vary the temperature of the annealing step according to primer binding sites and the choice of polymerase. Annealing temperature estimates can be found online at https://tmcalculator.neb.com. Increase the extension time for longer expected PCR products according to the manufacturer’s instructions.
- Add 2 μL of 6× loading dye to each sample and load 10 μL into a 1% agarose gel (or a 1.5% agarose gel for PCR products smaller than 1 kb) to perform gel electrophoresis. The recommended electrophoresis parameters are 120 V for 30 min.
- Run electrophoresis until the bands corresponding to inserted and uninserted products separate sufficiently (Fig. 5d).
- Using a clean blade or razor, cut out the top inserted band for two to three clones with no uninserted product visible, and gel extract using the QIAquick or MinElute gel extraction kits (Qiagen), following the manufacturer’s instructions.
- Confirm insertion, including orientation and distance from target sequence, by Sanger sequencing of extracted bands. Both pair of PCR primers (from Fig. 5a) can be used for sequencing.
  ◆TROUBLESHOOTING
- Inoculate 5 mL of LB cultures from grown spots made in Step 30 and incubate at 37 °C overnight (~12 h) for downstream applications.

#### (optional) Construct plasmid curing

● TIMING 2–3 d

▲**CRITICAL** While not required, we strongly recommend performing plasmid curing, especially for experimental applications that generate knockouts and require the generation of a stable line to study the potential phenotypic effect(s) of the induced manipulation. Failure to cure CAST plasmids could result in additional insertions (i.e., tandem insertions or off-target insertions), or potentially the excision of the integrated payload due to continued expression of transposase enzymes. To isolate clonal integrants that do not have a further risk of genetic heterogeneity, we recommend proceeding with this plasmid curing step.

▲**CRITICAL** Curing is not guaranteed to work across different plasmids and host cells, particularly for high-copy-number plasmids (such as pUC19 pDonors).

- Perform plasmid curing for clonal isolation before the start of the experiment by overnight growth at 37 °C if using pSPIN plasmids on the pSC101* temperature-sensitive backbone (option A) or by continued growth without antibiotic selection for different plasmid backbones (option B).
  - pSC101* plasmid curing:
    - Into a 50 mL conical tube, inoculate a cell spot from Step 30 in 15 mL of LB medium without spectinomycin selection and grow the culture overnight at 37 °C in a shaking incubator.
      ▲**CRITICAL STEP** Antibiotics other than spectinomycin should still be added to the growth medium if other plasmids or markers require active selection.
    - Dilute 2 μL of the overnight culture into 500 μL of LB medium in a microcentrifuge tube. Plate 100 μL of the same overnight culture onto an LB agar plate (no antibiotic supplemented).
    - Prepare two to three 1:10 serial dilutions of diluted culture from Step 39a(ii), each plated on a separate agar plate.
    - Incubate plates from Step 39a(ii–iii) at 37 °C overnight.
    - The following morning, inspect the plates and choose one where individual colonies are discernable. Pick five to six colonies and stamp each of them onto a new LB agar plate without spectinomycin and a new LB agar plate with 1× spectinomycin added. Number each stamp accordingly across the two plates.
    - Incubate the plates overnight at 37 °C.
      ▲**CRITICAL STEP** Stamped colonies growing only on the no-spectinomycin plate have had the pSC101* plasmid cured.
    - (Optional) Confirm plasmid loss by PCR. From the no-spectinomycin plate, pick five colonies, and screen as detailed in Steps 32–37. Suggested primers are in Supplementary Table 2.
      ▲**CRITICAL STEP** Cells with cured plasmids should show no visible amplification.
  - Curing procedure for other plasmid backbones.
    - Into a 50 mL conical tube, inoculate a cell spot from Step 30 in 15 mL of LB medium.
      ▲**CRITICAL STEP** LB medium should not contain any antibiotics selecting for backbones that need to be cured from cells.
    - Grow cells incubating culture overnight at 37 °C, in a shaking incubator. A short growth of 1–3 h at 42 °C increases curing efficiency.
    - Plate cells on selection LB agar plates and phenotypically characterize 20–30 colonies as in Step 39a(ii–vi).
    - Dilute 5 μL of the overnight culture from Step 39b(ii) into 15 mL of fresh LB medium, and incubate overnight at 37 °C.
      ▲**CRITICAL STEP** If curing more than one plasmid at once, colonies should be stamped on plates containing only one of the corresponding antibiotic types, in addition to plates without any antibiotics.
    - Isolate colonies growing only on the no-selection plate. If no such colony was observed, repeat Step 39b(iv) using the new overnight culture from Step 39b(iii). For multiple plasmids curing, if a colony was found without one of the target curing plasmids, inoculate from that colony and repeat Steps 39b(i–iv).
    - (Optional) Confirm plasmid loss by PCR. From the no-selection plate, pick five colonies, and screen as detailed in Steps 32–37. Suggested primers are given in Supplementary Table 2.
      ▲**CRITICAL STEP** Cells with cured plasmids should show no visible amplification.

### Part 5: DNA integration analysis

● TIMING 3–4 d

#### Extract gDNA from transformants or transconjugants in bulk and passage for clonal isolation

● TIMING 3–4 d

▲**CRITICAL** We recommend assessing integration efficiency across the entire population of cells as a quality control check for CAST activity. In addition, colonies at this stage will typically not be clonal after a single transformation step, and thus requires passaging repeatedly if the goal is clonal isolation.

- After incubation on LB agar from Step 25, obtain cells to conduct bulk gDNA extraction by selecting one of the two plates from each transformation that produced more than 100 colonies, but not a dense lawn of cells.
- Scrape a minimum of 100 colonies from the selected plate using a pipette tip or an inoculation loop, and fully resuspend by pipetting in 500 μL of LB medium.
  ▲**CRITICAL STEP** If very few colonies are produced from Step 25, they can be restreaked onto a new agar plate and incubated overnight to produce enough cell material for Step 40. If both plates produced dense lawns of cells from where individual colonies are not easily discernible, plate lower dilutions of the recovery. Dense lawns may reduce integration efficiency.
  ◆TROUBLESHOOTING
- Prepare 1:10 serial dilutions of cell resuspensions into 100 μL aliquots of LB medium and plate each dilution onto an LB agar plate containing the appropriate antibiotic selection and incubate overnight at 37 °C.
  ▲**CRITICAL STEP** This step is required for clonal isolation as discussed in the ‘DNA integration analysis’ section of the ‘Experimental design’. To perform clonal isolation, refer to Part 4: ‘Isolating clonal integrants by PCR’.
- (Optional) To increase integration efficiency, repeat Step 42 one to three times.
- Lyse cells and/or extract gDNA for PCR and qPCR analysis following the procedure in Boxes 7 and 8.

#### (optional) Population-wide PCR and qPCR analysis of transposon integration

● TIMING 3 h

▲**CRITICAL** While the primary scope of this protocol is the generation of clonal on-target integrants, population-wide PCR and qPCR analysis may be useful in troubleshooting or optimizing experimental conditions for transposition, especially for new target sites or target hosts, before proceeding to clonal integrant selection.

**Fig. 6 |:**
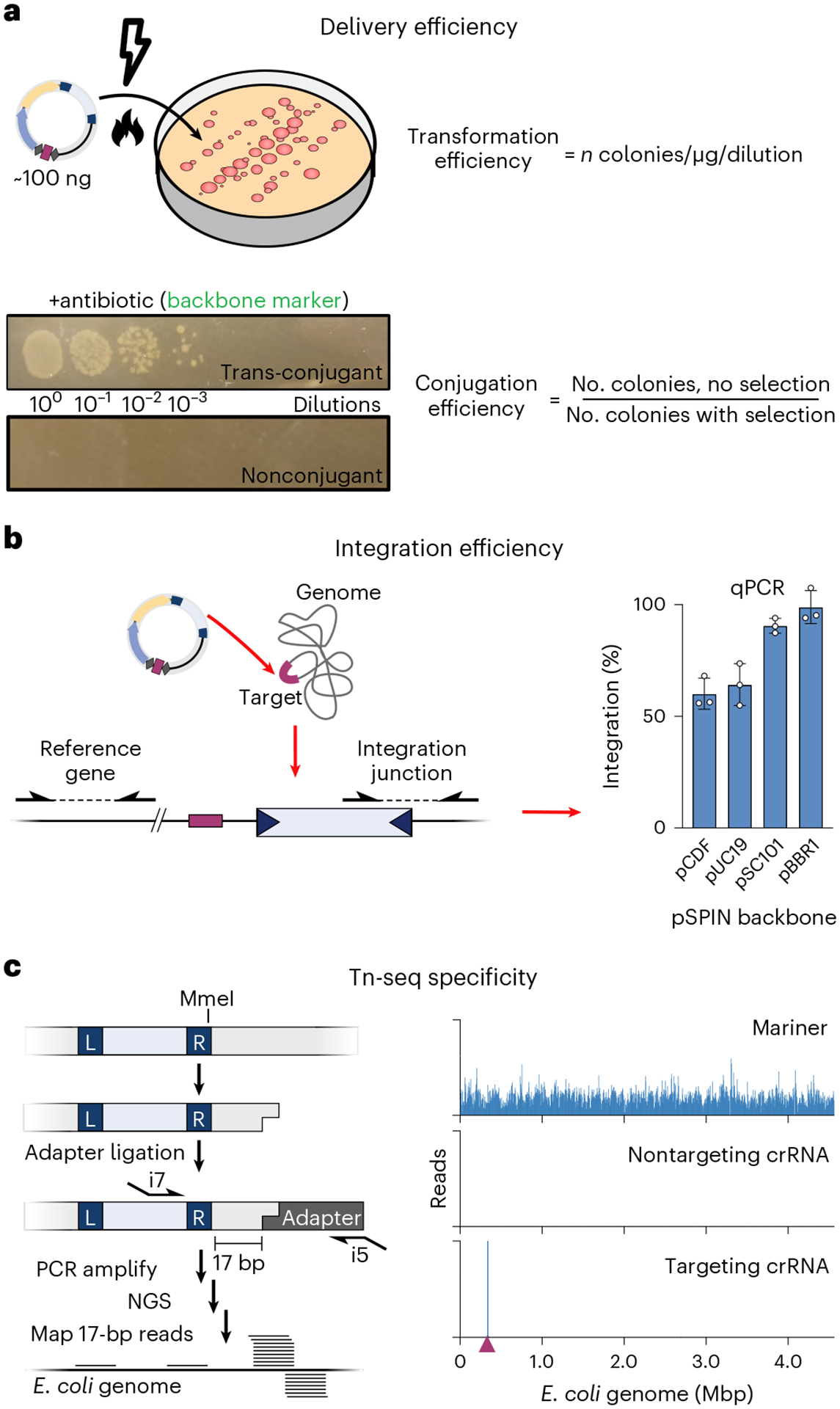
Anticipated results from a CAST editing experiment. **a**, Methods to assess delivery efficiency following transformation (i.e., chemical or electroporation) or conjugation. **b**, DNA integration efficiencies may be assessed and/or measured by PCR and/or qPCR. The graph on the right is a representative example of qPCR results obtained from experiments with pSPIN, with various backbones differing in their origin of replication/copy number that were performed in *E. coli* (right)^32^. **c**, To profile genome-wide specificity, Tn-seq may be applied to unbiasedly sequence all integration events within a sample of interest (left). The purple arrow indicates the target site. Representative data for the use of Mariner or VchCAST in *E. coli* are shown on the right, for both a nontargeting and targeting crRNA^33^. Images in **b** and **c** adapted with permission from ref. 32, Springer Nature America, Inc.

▲**CRITICAL** Albeit rarely, CAST-mediated transposition can result in off-target integration events. To estimate the genome-wide presence and frequency of off-target integration events, users may want to perform Tn-seq, a high-throughput sequencing method that sequences CAST transposon ends (Box 1). Specifically, our Tn-seq protocol is based on the generation of an MmeI mutation in the CAST right transposon end to allow for digestion, adapter ligation and subsequent PCR barcoding to sequence all sources of CAST insertions^32^ (Fig. 6). For wild-type Type I-F CASTs, we have observed that off-target integration is less than 1% of all insertion events^32^ and thus may not need to be profiled if the user’s goal is to generate a clonal functional knockout. For a more detailed Tn-seq protocol, please refer to our previous publication^32^. We have also developed a newer Tn-seq protocol based on tagmentation that does not require an MmeI mutation in the transposon ends^47^.

- Use the lysate or the extracted gDNA from Step 44 to confirm successful integration by performing population-wide PCR analysis (option A), or to confirm integration efficiency by qPCR (option B).
  - Population-wide PCR analysis
    ▲**CRITICAL** We recommend performing three PCR reactions for target site and sample: one pair each probing for the two possible insertion orientation (T-RL or T-LR), and a third control pair probing for an unmodified (wild-type) genomic locus.
    - Design primer pairs for PCR following the guidelines in Fig. 5a,b and Supplementary Table 2.
      ▲**CRITICAL STEP** We typically recommend using qPCR primers of 18–24 nt in length, with an average predicted melting temperature of 55 °C.
      ▲**CRITICAL STEP** It is best practice to test the amplification efficiency of each qPCR primer pair before use. This can be done by measuring the Cq values generated from a serial dilution series of the sample lysate or gDNA. Plots of Cq values versus log dilution should produce a straight line with a negative slope between −3.10 and −3.60, corresponding to a primer efficiency of >99%.
    - Set up a PCR reaction using the lysate or extracted gDNA (Step 44/Box7) as detailed below. Prepare a batch mastermix for multiple samples in parallel to minimize pipetting errors.

**Table:** 

Component	Amount (μL)	Final concentration
Lysate or gDNA (Step 44/Box 7)	5	
10 μM Primer-1 (Supplementary Table 2)	0.625	0.5 μM
10 μM Primer-2 (Supplementary Table 2)	0.625	0.5 μM
10 mM dNTPs	0.25	0.2 mM
Q5 Hot Start DNA Polymerase (NEB)	0.125	
5× Q5 Reaction Buffer	2.5	1×
MQ water (up to 12.5 μL)	3.375
      ▲**CRITICAL STEP** For genotyping, we recommend using a high-fidelity polymerase enzyme, such as the Q5 Hot Start DNA Polymerase (NEB). However, lower-fidelity polymerases can also be used.
      ▲**CRITICAL STEP** If only integration in the T-RL orientation is desired, there is no need to detect integration in the T-LR orientation. However, PCR to detect T-LR can be performed in parallel using a new pair of primers specific to the T-LR junction (Supplementary Table 2).
    - Perform the PCR reaction in a thermocycler using the following cycling parameters:

**Table:** 

Cycle number	Denature	Anneal	Extend	Hold
1	98 °C, 30 s	–	–	–
2–30	98 °C, 10 s	*X* °C, 20 s	72 °C, 30 s	–
31	–	–	72 °C, 2 min	–
32	–	–	–	4 °C
      ▲**CRITICAL STEP** Vary the temperature of the annealing step according to the primer binding sites and the polymerase enzyme used. The annealing temperature can be estimated using online calculators (tmcalculator.neb.com). For long PCR amplicons, increase the extension time according to the manufacturer’s instructions.
    - Add 2 μL of 6× loading dye to the PCR reaction and load 25 μL of the solution on a 1% agarose gel for gel electrophoresis. If the expected PCR products are smaller than 1 kb, we recommend using a 1.5% agarose gel.
    - After running the sample for 30 min at 120 V, visualize the gel on a UV transilluminator to confirm the presence of a band of the correct size, corresponding to a transposon–genome junction product.
      ◆TROUBLESHOOTING
    - (Optional) To further confirm the identification of a bona fide integration event, gel-extract the band of interest by using the QIAquick or MinElute gel extraction kits and confirm insertion by Sanger sequencing.
  - Population qPCR analysis
    - Design qPCR primers following the guideline in Fig. 5a,b and Supplementary Table 2.
      ▲**CRITICAL STEP** We typically recommend using qPCR primers of 18–24 nt in length, with an average predicted melting temperature of 55 °C.
      ▲**CRITICAL STEP** It is best practice to test the amplification efficiency of each qPCR primer pair before use. This can be done by measuring Cq values generated from a serial dilution series of the sample lysate or gDNA. Plots of Cq values versus log dilution should produce a straight line with a negative slope between −3.10 and −3.60, corresponding to a primer efficiency of 99%.
    - Prepare a mixture of the forward (10 μM) and the reverse primer (10 μM) by adding 10 μL of each (Supplementary Table 2) to 380 μL of EB buffer or MQ water.
    - In a 384-well qPCR plate, set up a qPCR reaction for each diluted lysate as described below. Preparation of a batch mastermix is recommended for multiple samples in parallel.

**Table:** 

Component	Amount (μL)	Final concentration
Lysate or gDNA (Step 44/Box 7)	2	
Diluted primer mix (10 μM)	2	5 μM
SsoAdvanced Universal SYBR Green Supermix	5	
MQ water (up to 10 μL)	1
      ▲**CRITICAL STEP** For each sample, perform qPCR with primer pair RL and pair G (Supplementary Table 2). If integration efficiency in the T-LR orientation is needed, perform a third parallel qPCR reaction with primer pair L. (Optional) Perform each reaction in three separate technical replicates. We strongly recommend including crRNA-nontargeting and water-only samples as negative controls.
    - Run the qPCR reaction in a 384-well qPCR thermocycler with the following conditions:

**Table:** 

Cycle no.	Denaturation	Annealing and extension
1	98 °C, 2:30	–
2–40	98 °C, 10 s	62 °C, 20 s
      ▲**CRITICAL STEP** Vary the annealing and extension for new primer pairs as needed to obtain sufficiently high amplification efficiencies, without background off-target amplifications.
    - Calculate estimated DNA integration efficiency as in Fig. 5a,b and Box 8.
      ◆TROUBLESHOOTING
    - (Optional) Add 2 μL of 6× loading dye to each well and load 25 μL of each sample of a 1.5% agarose gel to perform gel electrophoresis. Typical electrophoresis conditions for a ~200 bp product are 130 V for 22 min.
      ◆TROUBLESHOOTING
    - (Optional) Excise bands at the expected sizes for reactions RL and LR, and extract the DNA using the MinElute Extraction Kit (Qiagen). Elute the DNA in 10 μL of EB buffer for best results.
    - Confirm sequence by Sanger sequencing using one of the primers in the primer pair.

## Troubleshooting

Troubleshooting advice can be found in Table 1.

## Timing

### Part 1

Step 1, Target selection and crRNA design: 3 h

### Part 2

Steps 2–24, Generation of custom crRNAs and payloads: 2 d

Steps 2–23, Cloning custom crRNA spacers: 2 d

Step 24, Cloning custom transposon DNA (optional): 2 d

### Part 3

Steps 25–28, Delivery into cells: 3–4 d

Step 25, Perform transposition and conjugation in target strain: 3–4 d

Steps 26–28, Estimation of conjugation efficiency: 1 h

### Part 4

Steps 29–39, Culturing, selection and/or curing: 3–4 d

Steps 29–38, Isolating clonal integrant by PCR: 18 h

Step 39, Construct plasmid curing: 2–3 d

### Part 5

Steps 40–45 DNA integration analysis: 6–7 d

Steps 40–44, Extract gDNA from transformants or transconjugants in bulk and passage for clonal isolation: 3–4 d

Step 45, Population-wide PCR and qPCR analysis of transposon integration: 3 h

## Anticipated results

The use of this protocol should allow the generation of clonally integrated mutant bacterial strains that can be used for various applications (Fig. 3). We encourage systematic analyses to carefully assess the efficiency of both, plasmid delivery and DNA integration, as well as optional analyses to interrogate genome-wide specificity by Tn-seq (Fig. 6). Regardless of the delivery method, transformation efficiency can be straightforwardly calculated by dividing the number of resulting colonies with vector backbone selection by the input amount of vector (20–100 ng recommended), taking the dilution factor used for plating into account (Fig. 6a, top). When conjugation is used as the delivery method and plasmids are stably maintained in recipient cells, efficiency can be calculated via selective plating on the plasmid vector backbone marker in comparison to plating without any selectable marker (Fig. 6a, bottom). The ratio of the number of colonies obtained under antibiotic selection versus no selection provides an estimate of how efficiently the plasmid was conjugated from the donor to the recipient strain.

For a typical DNA integration reaction, efficiency is normally in the range of 50–99% for ~1 kb payloads in *E. coli* and other bacteria (Fig. 6b), as estimated via qPCR of the integration junction and a reference gene locus (for primer considerations, see Fig. 5). Excitingly, we have observed that the integration efficiency can be further increased either by conducting transposition and growth at lower temperatures (30 °C)^32^ or through the use of higher-activity homologous CAST systems^41^. Lastly, while beyond the scope of this protocol, it may be helpful to perform whole-genome sequencing on isolated clones to further confirm the genotype, as well as confirm the absence of off-target insertions, particularly when aiming at multiple genomic insertions of the transposon during strain engineering. In previous studies, we performed Tn-seq using modified mini-Tn substrates with an MmeI digest site, to unbiasedly report on all genomic regions containing a mini-Tn insertion by high-throughput sequencing^31,33^ (Fig. 6c). After sequencing Tn-seq libraries, we recommend to follow a computational pipeline for detecting off-target events^32,33^ that filters for reads that contain an intact transposon right end, extracts the DNA sequence (17–20 bp) flanking the transposon end, maps these flanking sequences to the genome of the target bacteria, and finally visualizes the number of genome-mapping reads to assess off-target events.

**Fig. 7 |:**
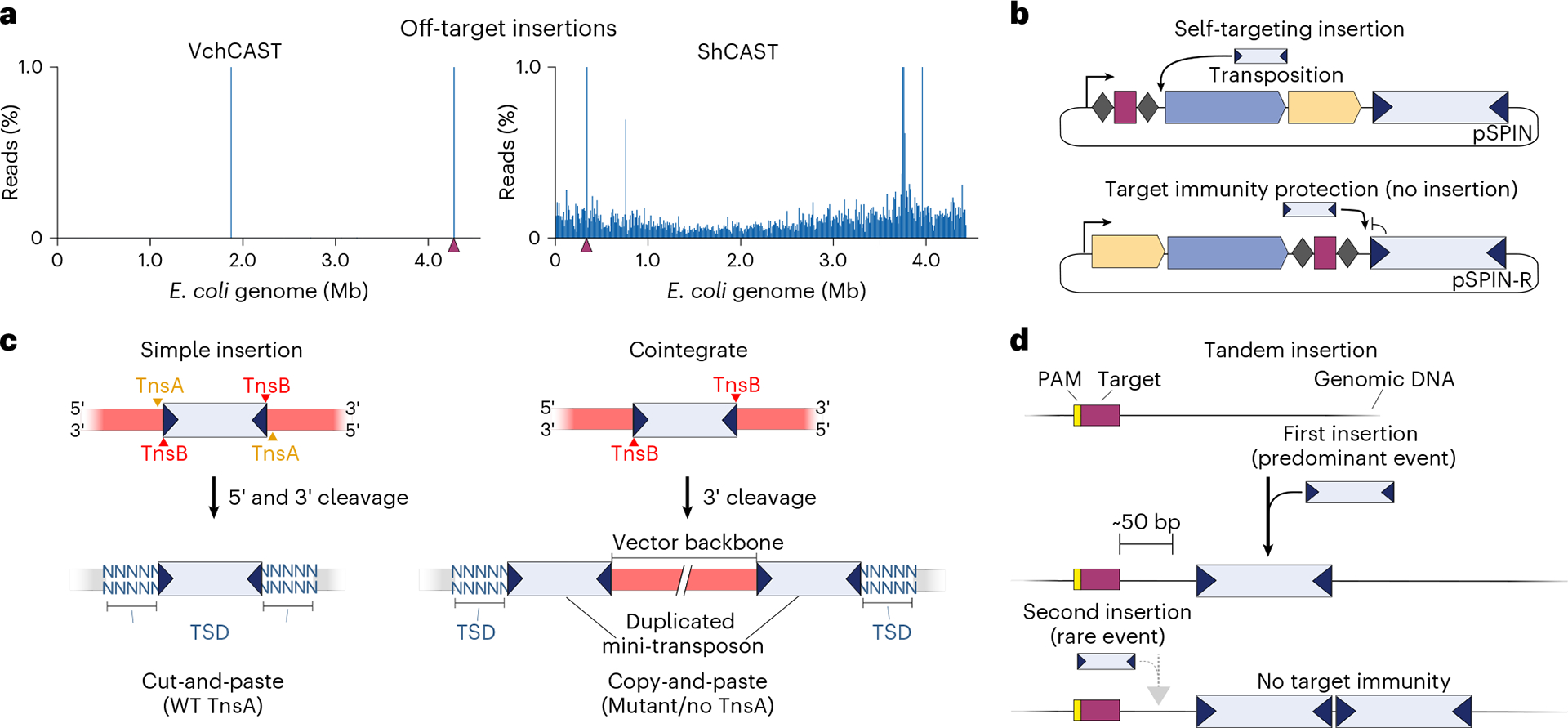
Alternative integration byproducts. **a**, Comparison of genome-wide specificity between VchCAST (Type I-F) and ShCAST (Type V-K), as assessed via random fragmentation-based NGS library preparation, focused on reads comprising 1% or less of genome-mapping reads. The purple arrow indicates the target site. The Type I-F system exhibits exquisite accuracy with low off-target insertions, whereas both Type V-K systems exhibit rampant, off-target integration across the *E. coli* genome. **b**, A depiction of undesirable self-targeting insertion events that can occur downstream of the CRISPR spacer due to flexible PAM recognition, and a redesigned vector (pSPIN-R) that ablates self-targeting integration products by harnessing the mechanism of target immunity. **c**, Type I-F CASTs can generate simple insertion products via nonreplicative, cut-and-paste transposition (left), whereas Type V-K CASTs lacking TnsA (or Type I-F CASTs with inactivated TnsA) generate cointegrate products via replicative, copy-and-paste transposition (right). Cointegrates contain two copies of the inserted mini-Tn that flank the vector backbone. **d**, A depiction of low-frequency tandem payload insertion events in Type I-F CASTs.

**Fig. 8 |:**
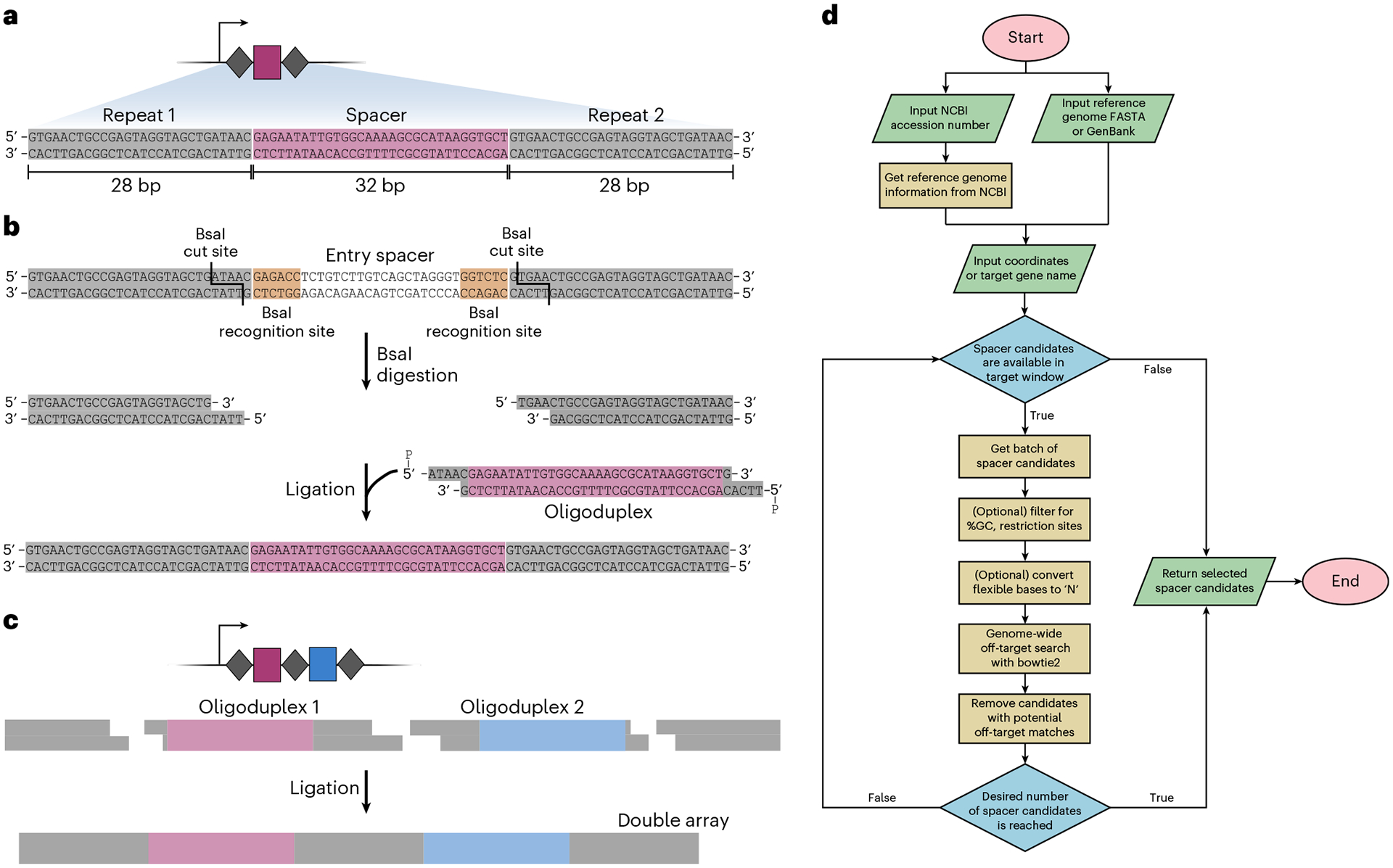
Cloning CRISPR spacers for CAST systems. **a**, A diagram of a single-spacer CRISPR array for VchCAST, characterized by two 28-bp repeats flanking a 32-bp spacer complementary to the target site. **b**, Strategy to clone spacers into expression vectors using BsaI restriction digestion and oligoduplex ligation. **c**, Example of a cloning strategy to generate multispacer CRISPR arrays, for multiplexed RNA-guided DNA insertions. **d**, A diagram of the computational methodology for the CAST guide RNA tool. Image **d** adapted with permission from ref. 32, Springer Nature America, Inc.

This experimental approach revealed that the VchCAST system typically integrates DNA payloads with >99% on-target specificity, as compared with the frequent nonspecific insertions generated by both Mariner transposons and Type V-K CAST systems^32,33,41^ (Fig. 7 and Box 1). In addition, long-read sequencing on either the PacBio or Nanopore platforms can also confirm integration product purity (simple insertion versus cointegrate, Fig. 7 and Box 1) and specificity through individual reads spanning the entire transposon insertion^45,46^. However, since the high target specificity of Type I-F CAST systems are well documented with proper crRNA design^32,33,35,39^, many engineering applications may not require genome-wide analyses of transposition events, and thus protocols for long read sequencing are not detailed here. Finally, armed with this protocol, we anticipate that the application of this protocol will expand the bacterial engineering toolkit to include, among others, (1) multiplexed open reading frame (ORF) disruption using multispacer CRISPR arrays, by cloning in multiple crRNA duplexes (Fig. 8 and Box 2), in conjunction with gRNA libraries to determine gene essentiality under various environmental conditions; (2) iterative gain-of-function knock-ins of metabolic operons using orthogonal CAST systems that circumvent target immunity (i.e., homologous Type I-F CASTs, or Type I-F and Type V-K CASTs); and (3) multiplexed editing (Fig. 8 and Box 2) of multiple target bacteria in complex consortia to study the genetic interactions between microorganisms in communities.

## Supplementary Material

2024_Gelsinger_Nature Protocols_SupplementaryTables.xlsx

## Figures and Tables

**Table 1 |:** Troubleshooting table

Step	Problem	Possible reason	Solution
20	Recombination between CRISPR repeats	Nuclease degradation of digested plasmid	Avoid using excessive amounts of restriction enzyme in each reaction. Replace water, buffer and enzyme stocks to mitigate risk of DNase contamination
		Inefficient ligation	Repeat the preparation of 5’-phosphorylated oligoduplex substrates
		Suboptimal conditions for ligation	Systematically test and optimize the relative amounts of digested vector and oligoduplex insert
		Erroneous ligation	Prepare ligation mixture on ice and add ligase to the reaction mixture last
20, 24a(x), 24b(vi)	High rates of background (i.e., circularized parental vector)	Too much digested vector plasmid	Repeat ligation or Gibson assembly using decreased amounts of digested vector. Increase digestion time or perform overnight digestion. Screen more colonies by colony PCR
20, 24b(vi)	No colonies on ligation cloning plates	Insufficient amounts of digested vector	Repeat ligation using increasing amounts of digested vector
		Inefficient ligation or transformation	Ensure phosphorylation and annealing of primers were done properly. Repeat ligation at room temperature for 2 h to overnight. Use commercial chemically competent cells or electrocompetent cells to increase transformation efficiency
24a(x)	No colonies on Gibson cloning plates	Insufficient amounts of digested vector or PCR insert	Repeat ligation using increased amounts of digested vector and PCR insert, and systematically optimize the relative ratio
		Inefficient assembly or transformation	Incubate Gibson assembly reaction for longer (4 h to overnight). Use commercial chemically competent cells or electrocompetent cells to increase transformation efficiency
25, 25c(ii), 41	Few or no colonies on transposition plates	Inefficient transformation	If initial experiments involved a cotransformation with both pEffector and pDonor, we suggest instead transforming cells with pDonor only, preparing competent cells with the resulting transformant, and then delivering pEffector in a subsequent transformation step. Repeat competent cell preparation carefully. Increase plasmid amount and competent cell volume during transformation. Perform electroporation instead of chemical transformation
		Cellular toxicity from the vector construct	Replace the strong constitutive vector driving expression of constructs with weaker or inducible promoters
		Plasmid backbone unsuitable for target cells	Replace the vector backbone with a vector or shuttle vector that has been validated for the target species and/or target strains
37	Noisy Sanger sequencing chromatograms	Isolated clone may be polyclonal and contain multiple insertion orientations and/or integration distances	If the chromatogram is not improved by improving quality of Sanger sequencing sample, streak the clone onto solid media to obtain new colonies, then repeat PCR analysis and Sanger sequencing on these new clones
45a(v), 45b(vi)	No PCR/qPCR bands detected	Suboptimal conditions for transposition	Optimize incubation temperature during overnight incubation in Step 15. Avoid plating cells at a high density. Repeat Steps 16 and 17 for one or several additional nights of growth
		Recombination or degradation of vector constructs	Extract plasmid DNA from cells and sequence by Sanger or NGS. If recombination occurred within repetitive regions, resulting in deletion of machinery genes, modify the vector to eliminate these repeated regions
		Successful transposition is toxic to cells	Redesign CRISPR target and spacer sequences
		Unsuitable primers	Redesign primers and optimize annealing temperature by running a gradient of annealing temperatures in parallel
		Plasmid backbone unsuitable for target cells	Replace the vector backbone with a vector or shuttle vector that has been validated for target species or strains of choice
45b(v)	Low transposition efficiencies	See troubleshooting for Steps 24 and 25 above	See troubleshooting for Steps 23 and 25 above

## Data Availability

Next-generation sequencing (NGS) data used for Figs. 6 and 7 are available in the National Center for Biotechnology Information (NCBI) Sequence Read Archive (BioProject accession code PRJNA668381). Published genomes used for Tn-seq analyses in Fig. 6 were obtained from the NCBI (accessions codes CP001509.3).

## Key points

- The protocol describes a novel and versatile CRISPR-associated transposase (CAST) system for the targeted and precise insertion of large DNA payloads into bacterial genomes.
- Compared with pre-existing methods, this approach allows single and multiplexed insertion events at a desired location, with increased efficiency, reduced population heterogeneity, and improved specificity.

## Box 1 Alternative integration byproducts

Although Type I-F CAST systems robustly generate single-copy simple insertion products^32,33,46^, alternative and/or undesired integration products are also possible with CAST systems (Type I-F/Type V-K), including off-target insertions (Fig. 7a), self-inactivating vector insertions (Fig. 7b), on-target cointegrate products (Fig. 7c), and tandem on-target insertions^45,56,57,59^ (Fig. 7d). Off-target insertions can arise through both RNA-dependent and RNA-independent processes, and appear to be much more prevalent (Fig. 7a) for Type V-K CAST systems (e.g., ShCAST) than for Type I-F CAST systems (e.g., VchCAST), with our recent study highlighting key molecular details of this behavior^47^.

We previously reported that QCascade-directed DNA integration exhibits a high degree of PAM promiscuity, including low activity with the mutant 5′-AC-3′ self-PAM present within the CRISPR repeats flanking the spacer. Thus, low-level targeting of the spacer found within the CRISPR arrays can lead to self-targeting insertions that inactivate the pSPIN vector, as detected in the majority of our *E. coli* Tn-seq datasets^32^ (Fig. 7b). To circumvent this undesired self-targeting product, we redesigned the expression vector such that the CRISPR array is positioned in close proximity to the mini-Tn itself. As such, it becomes largely protected from self-targeting due to the mechanism of transposon target immunity. These modified pSPIN-R vector completely abrogated self-targeting, at least under the conditions tested (Fig. 7b). We therefore encourage users to carefully monitor background levels of vector inactivation due to self-targeting, and to consider use of pSPIN-R vectors instead.

Simple insertion products are generated by nonreplicative, cut-and-paste transposition events. These events rely on the activity of a TnsA endonuclease and a TnsB transposase for 5′- and 3′-end cleavages at the donor site, respectively, leading to excision of the transposon DNA as a double-stranded DNA molecule. The TnsB enzyme subsequently catalyzes strand-transfer reactions at the target site using both 3′ ends of the transposon, followed by gap repair to produce the final integration product flanked by a 5-bp TSD (Fig. 7c). Inactivating mutations in the TnsA active site result in the formation of so-called cointegrate products. Cointegrates arise from replication-dependent, copy-and-paste transposition, and are characterized by the presence of two copies of the transposon flanking the entire donor vector backbone (Fig. 7c). Most notably, Type V-K CAST systems lack TnsA altogether and exclusively generate cointegrate products, although these products can resolve to simple insertions through HR^45,46,56,57^. We and others have applied PCR-free long-read sequencing to unbiasedly characterize and quantify both types of integration products, demonstrating that Type I-F CAST systems show optimal product purity, but we also encourage interested readers to consider engineered Type V-K CAST systems (HELIX) that show improved properties for both transposition pathway and specificity. Lastly, both types of CAST systems can also generate tandem insertions (Fig. 7d), since the target site complementary to guide RNA is not destroyed during the integration reaction itself. The frequency of tandem insertions^55^ is naturally limited through the mechanism of target immunity^32,45,46,53,77^, and can be further reduced by restricting the duration of CAST expression.

## Box 2 Cloning CRISPR spacers for CAST systems

The first step in CAST guide RNA design is to identify available PAMs that would place the desired transposon integration site 48–50 bp downstream of the adjacent 32-bp target. The VchCAST systems (Tn6677) recognizes 5′-CN-3′ PAMs, whereas other homologous Type I-F CAST systems are even more permissive^41^, thus offering a flexible targeting window. CRISPR arrays encoding the candidate crRNA guides, flanked by two repeats (Fig. 8a), should be designed so that the 32-bp spacer sequence exactly matches the 32 bp immediately at 3′ of the PAM on the target genome (Fig. 1d). crRNA spacers with a high degree of sequence identity to other regions of the genome should be filtered out to minimize the risk of off-target insertions. Once these requirements are met, the crRNA can be cloned into the appropriate CAST vector (Fig. 8a–c). We encourage users to use our in-house pipeline available on Github (https://github.com/sternberglab/CAST-guide-RNA-tool) to screen for candidate crRNA using a BLAST-based approach (Fig. 8d).

Once the final spacer sequences have been chosen, they can be cloned via ligation into pEffector or pSPIN entry vectors, in which Type II-S restriction sites (BsaI or BbsI) are flanked by two CRISPR repeats (Fig. 8b). A pair of oligonucleotides should be designed as depicted in Fig. 8b. In particular, oligo-1 should contain 5 nt of overlap with the plasmid digestion site at the 5′ end, followed by the 32-nt spacer sequence, and ending in 1-nt overlap with the plasmid digestion site at the 3′ end. Oligo-2 instead should contain the reverse 32-nt spacer, again with a 5-nt overlap at the 5′ end and a 1-nt overlap at the 3′ end. Both oligos are hybridized together to form a sticky-end DNA product, containing the crRNA spacer of interest, ready for ligation into pEffector or pSPIN receiving vectors, previously digested with the relevant restriction enzyme (BsaI or BbsI, respectively).

Multiplexed CRISPR arrays, which encode distinct crRNAs to enable targeted insertion at multiple genomic sites, can also be constructed following a similar design and cloning strategy. In this case, rather than using a single pair of hybridized oligos, multiple oligoduplexes are combined into a single ligation reaction that yields the desired CRISPR array (Fig. 8c).

## Box 3 In-house 5′ phosphorylation of oligoduplex

● TIMING 1.5 h

### Procedure

1. Prepare the reaction mix to phosphorylate the desired oligonucleotides at their 5′-end as detailed below:
  **Table:**
  Component	Amount (μL)	Final concentration
  Oligo-1 (100 μM) Supplementary Table 3	2.5	10 μM
  Oligo-2 (100 μM) Supplementary Table 3	2.5	10 μM
  T4 Polynucleotide Kinase (NEB)	0.5	
  10× T4 DNA Ligase buffer (NEB)	2.5	1×
  MQ water (to a final volume of 25 μL)	17	
2. Incubate the reaction mix at 37 °C for 30 min.
3. Stop the reaction by performing heat inactivation at 65 °C for 20 min.
4. Heat the mixture to 95 °C for 2 min.
5. Allow to gradually cool to room temperature.

## Box 4 Generation of chemically competent EcGT2 donor strain harboring pSPIN by chemical transformation

● TIMING 2 d

### Procedure

▲**CRITICAL** Prepare Buffer A (80 mM MgCl_2_, 20 mM CaCl_2_) and Buffer B (100 mM CaCl_2_, 16% glycerol), and get them ice-cold before beginning the protocol.

1. Inoculate 10 mL of LB medium with a single colony of EcGT2 cells from a LB-agar plate or a frozen glycerol stock and grow the culture at 37 °C overnight, under constant agitation.
  ▲**CRITICAL** Supplement the medium with 1× concentration of the appropriate antibiotic, if required for the target strain.
2. The following day, using a 250 mL sterile baffled Erlenmeyer flask, make a 1:100 dilution of the overnight culture into 100 mL of LB medium supplemented with antibiotics if needed.
  ▲**CRITICAL** A 100 mL culture produces enough competent cells for ~80 50 μL transformation reactions. Scale up the volume (up to 1 L of medium) if larger preparations are required.
3. Incubate with shaking (120 rpm) at 37 °C and measure OD_600_ of the culture every 30 min to 1 h.
4. Once the cells have reached an OD_600_ of ~0.5 (approximately after 1–2.5 h), make 50 mL aliquots of culture in 50 mL conical tubes, and incubate all tubes on ice for 15 min.
  ▲**CRITICAL STEP** From this point onwards, cells should be kept on ice as much as possible.
5. Pellet the cells by centrifuging the samples for 10 min at 4,000*g* at 4 °C and discard the supernatant.
6. Add 20 mL of ice-cold Buffer A (80 mM MgCl_2_, 20 mM CaCl_2_) into each conical tube and resuspend pellet fully by pipetting up and down.
7. Centrifuge the samples as in step 5.
8. Add 2 mL of ice-cold Buffer B (100 mM CaCl_2_, 16% glycerol) into each conical tube and resuspend the pellets fully by pipetting.
9. Combine cell resuspensions from multiple tubes into one tube.
10. Add 70 μL DMSO every 2 mL of resuspension. Mix gently by pipetting and incubate on ice for 15 min. Meanwhile, place PCR strip tubes or microcentrifuge tubes on ice.
11. After this incubation step, add an additional 70 μL of DMSO every 2 mL of resuspension and mix gently by swirling the tube.
12. Aliquot 100 μL of cell resuspension into cold PCR strip tubes.
13. Transform cells immediately (step 14 below), or flash freeze them in liquid nitrogen for long-term storage at −80 °C for up to 12 months.
  ▲**CRITICAL** Freezing cells usually decreases transformation efficiency.
14. On ice, pipette 20–100 ng of each CAST plasmid construct (pSPIN) with 50 μL of chemically competent EcGT2 cells and mix cells by swirling the tube.
15. Incubate on ice for 15 min, heat shock at 42 °C for 30 s, and return the tubes on ice for 5 min.
16. Add each transformation to a microcentrifuge tube containing 950 μL of LB medium. Recover with shaking (120 rpm) at 37 °C for 1 h.
17. Plate 100 μL of recovery on an LB agar plate containing the appropriate antibiotic and DAP (Supplementary Table 1).
18. Pellet the remaining 900 μL of recovery by centrifugation at 4,000*g* for 5 min, discard the supernatant and resuspend the pellet in 100 μL of LB medium before plating the cell suspension on a LB agar plate supplemented with the appropriate antibiotic. Incubate plates at 37 °C overnight.
19. The following day, pick five colonies of EcGT2 pSPIN transformants and inoculate each colony separately in 5 mL LB liquid medium supplemented with appropriate antibiotic and DAP.
20. Grow the culture overnight at 37 °C, under constant agitation.
21. Repeat step 19 for the recipient strain without supplementing DAP. Now that the donor strain harboring a CAST plasmid of interest has been generated, delivery of pSPIN from the donor into the recipient strain for subsequent editing can be carried out as detailed in Box 5.

## Box 5 Procedure for the conjugative delivery of pSPIN

● TIMING 3 d

### Procedure

1. Pipet 1.5 mL of EcGT2 donor strain harboring a CAST plasmid of interest from Box 4 into a 1.7 mL microcentrifuge tube. Do the same for the desired recipient strain in a separate microcentrifuge tube.
2. Spin down the cells in a centrifuge for 5 min, at 4,000*g* at room temperature.
3. Aspirate the supernatant by pipette and aliquot another 1.5 mL of donor or recipient on top of the remaining pellet in each respective tube. Resuspend the pellet by pipetting up and down.
4. Centrifuge the samples for 5 min at 4,000*g* at room temperature.
5. Aspirate the supernatant by pipette and wash the pellet by adding 1 mL of 1× PBS pH 7.4 to each tube.
6. Spin down aliquoted cultures by centrifuging for 5 min at 4,000*g* at room temperature.
7. Repeat steps 5–6 for a total of three times.
8. After the final wash, resuspend donor and recipient pellets in 1 mL of 1× PBS pH 7.4.
9. Calculate number of cells either by OD_600_ or flow cytometry-based count.
  ▲**CRITICAL** Aim to have ~ 8 × 10^8^ to 1 × 10^9^ cells (*E. coli* OD_600_ of 1) for both, donor and recipient cells, to have a final 1:1 ratio. If the recipient is recalcitrant to conjugation, it is recommended to increase the donor:recipient ratio to 10:1, to increase conjugation efficiency.
10. Set up the conjugation reaction in a new 1.7 mL microcentrifuge tube, by pipetting the required volumes corresponding to the adequate number of cells for both donor and recipient, in the same reaction tube.
  ▲**CRITICAL** If conjugation volumes exceed >1.7 mL of culture solution, it is possible to further concentrate the cells by centrifugation (step 6) and resuspend in a smaller volume of 1× PBS pH 7.4.
  ▲**CRITICAL** In parallel, set up negative control reactions by pipetting the same number of either donor or recipient cells into individual 1.7 mL microcentrifuge tubes.
11. Spin all tubes down in a centrifuge for 5 min at 4,000*g* at room temperature.
12. Carefully aspirate all supernatant and discard, leaving behind only a cell pellet.
13. Carefully resuspend each pellet by pipetting in 10 μL of 1× PBS pH 7.4.
  ▲**CRITICAL STEP** Be sure not to introduce any bubbles in the mixture, as the solution will be highly viscous.
14. Pipet 10 μL of the cell mixture as a uniform spot onto LB plates (one spot per plate) supplemented with DAP.
  ▲**CRITICAL STEP** Do not introduce any bubbles in the spot as this will disrupt conjugation (cell-to-cell contact). In addition, different media conditions (non-LB) should be considered here, depending on the recipient strain.
15. Allow spots to dry for up to 30 min by incubating the plates at room temperature.
16. Incubate all plates at 37 °C for 16–24 h.
  ▲**CRITICAL STEP** A longer time for conjugation can increase the conjugation efficiency.

## Box 6 Selection of positive transconjugants

● TIMING 1–2 d

### Procedure

1. After 16–24 h of growth, spot reactions from Box 5 will have fully grown.
2. Scrape each individual spot reaction with an inoculation loop and swirl it into 1 mL of 1× PBS pH 7.4 in a 1.7 mL microcentrifuge tube.
3. Carefully resuspend the cells by fully pipetting the solution with a P1000 pipet.
4. In a 96-well plate, make serial dilutions (from 10^0^ to 10^−7^) of each conjugation samples as well as the donor-only and the recipient-only controls, by mixing 20 μL of sample with 180 μL of 1× PBS pH 7.4 as detailed below.
  **Table:**
  Amount 1x PBS pH 7.4 (μL)	Amount sample (μL)	Final concentration
  0 μL	200 μL	10^0^
  180 μL	20 μL of a 10^0^ dilution	10^−1^
  180 μL	20 μL of 10^−1^	10^−2^
  180 μL	20 μL of 10^−2^	10^−3^
  180 μL	20 μL of 10^−3^	10^−4^
  180 μL	20 μL of 10^−4^	10^−5^
  180 μL	20 μL of 10^−5^	10^−6^
  180 μL	20 μL of 10^−6^	10^−7^
5. Thoroughly mix by pipetting the dilutions using a p200 multichannel pipette.
6. For each sample, plate one 5 μL spot of each dilution.
7. Using an eight-well multichannel pipette onto LB-agar plates without antibiotics or LB-agar plates supplemented with the appropriate antibiotic selection.
  ▲**CRITICAL STEP** If very low efficiency is expected, spin down the conjugation 5 min at 4,000*g* at room temperature, resuspend by pipetting in 100 μL and plate all.
  ▲**CRITICAL STEP** Do not supplement DAP in any of the plates used for selection of the transconjugants. DAP will allow for the growth of the donor strain and convolute downstream results downstream.
8. Allow the spots to dry for up to 30 min by incubating the plates at room temperature.
9. In addition, plate 100 μL of resuspended conjugation reactions from Box 6, step 3 on an LB agar plate containing appropriate antibiotic selection. These plates will be used for acquiring biomass to estimate integration efficiency.
10. Incubate all plates at 37 °C for 12–24 h.

## Box 7 Cell lysis and extraction of gDNA for downstream applications

● TIMING 3 h

### Procedure

▲**CRITICAL** The cell suspension has roughly equivalent cell material as a turbid overnight culture and can be used for gDNA extraction using the Wizard gDNA Purification kit or equivalent. However, for general PCR/qPCR, it is sufficient to extract DNA by supplying heat to lyse the cells as described below.

1. Add 100 μL of resuspended cells from Steps 42 or 43 into 900 μL of LB medium in a plasmid cuvette, and measure OD_600_.
2. Dilute cell suspension or scrape and resuspend more colonies until the OD_600_ reaches ~0.4–0.5.
3. Pipette 170 μL of each cell resuspension into a microcentrifuge tube. If many parallel resuspensions are involved, PCR strip tubes or PCR plates can be used instead, with 85 μL of resuspension per tube or well.
4. Pellet the cell suspension by centrifuging microcentrifuge tubes at 12,000*g* for 2 min at room temperature, or at 4,000*g* for 5 min if using PCR strips or PCR plates.
5. Discard the supernatant and fully resuspend the pellet in 160 μL (or 80 μL if using PCR strips or PCR plates) of MQ water by gently pipetting up and down.
6. Heat the samples at 95 °C for 10 min using a heat block or thermocycler.
  ▲**CRITICAL** If using microcentrifuge tubes, seal the caps with parafilm or plastic cap lock.
7. Cool the samples to room temperature and pellet down by centrifuging at 4,000*g* for 5 minutes.
8. Transfer the supernatant into a new 1.5 mL tube.
9. Dilute 10 μL of each supernatant into 390 μL of MQ water in a new microcentrifuge tube. If using PCR strip tubes or PCR plates, dilute 2 μL into 78 μL of MQ water into new PCR strips or PCR plates.
  ■**PAuSE PoINT** Diluted lysates can be stored for several months at −20 °C.

## Box 8 qPCR-based calculation of DNA integration efficiencies

Integration efficiency measurements made for a given insertion orientation (e.g., T-RL) can be by qPCR using Cq values obtained from a primer pair ‘RL’ probing for the desired orientation, and Cq values of a primer pair ‘G’ probing for a reference genomic locus, as follows:

$$\%T-\text{RL}=100\times 2^{\text{Cq}\left(\text{Pair}\;\text{G}\right)-\text{Cq}\left(\text{Pair}\;\text{RL}\right)}$$

Similarly, the percentage integration in the T-LR orientation can be calculated using the Cq value for the T-LR primer pair. The total integration efficiency at the target site is then calculated as the sum of the two integration efficiencies, in the T-RL and T-LR orientations. Importantly, using this method, the error magnitude (i.e., PCR amplification efficiency between different loci) increases as the efficiency approaches 100%, and it is possible to obtain artifactual values larger than 100%. In such scenarios, we recommend testing multiple different primer pairs and/or repeating the qPCR with additional biological and technical replicates.

A more definite measurement of integration efficiency can be obtained by normalizing against a clonal integrant. Cells containing clonal insertions, which theoretically have an integration efficiency of 100%, in one orientation should first be isolated (Step 45). qPCR analyses are then performed as above on both the clonal strain and the unknown sample(s), followed by normalization. For example, the T-RL integration efficiency can be calculated as:

$$\text{True}\%T-\text{RL}=100\times \left(T-\text{RL}\;\text{sample}\right)/\left(T-\text{RL}\;\text{clonal}\right)$$
